# Supplementary material for: Functional specificity of recurrent inhibition in visual cortex
Source: Neuron. Author manuscript; Available in PMC 2025 Nov 4. (PMC7618320; doi:10.1016/j.neuron.2023.12.013)
Supplement: Supplementary Material [file EMS209709-supplement-Supplementary_Material.zip › 1-s2.0-S0896627323009728-mmc2.pdf]

Neuron

Article

Functional specificity of recurrent inhibition in visual cortex

Highlights

- PV+ neurons strongly inhibit those neurons that provide them with strong excitation
- PV+ neurons in V1 have broadly tuned but heterogeneous responses to visual stimuli
- Visual responses of PV+/pyramidal neurons predict the strength of their connections

Authors

Petr Znamenskiy, Mean-Hwan Kim, Dylan R. Muir, M. Florencia Iacaruso, Sonja B. Hofer, Thomas D. Mrsic-Flogel

Correspondence

petr.znamenskiy@crick.ac.uk (P.Z.), t.mrsic-flogel@ucl.ac.uk (T.D.M.-F.)

In brief

Znamenskiy et al. reveal unexpected precision of synaptic strength of parvalbumin-positive inhibitory neurons in the mouse visual cortex. By measuring their visual responses and synaptic connectivity, they show that PV+ cells preferentially inhibit those pyramidal cells that provide them with strong excitation and have similar patterns of responses *in vivo*.

Znamenskiy et al., 2024, Neuron *112*, 991–1000
March 20, 2024 © 2023 The Author(s). Published by Elsevier Inc.
https://doi.org/10.1016/j.neuron.2023.12.013

CellPress

Neuron

CellPress
OPEN ACCESS

Article

Functional specificity of recurrent inhibition in visual cortex

Petr Znamenskiy,[1,2,3,4,9,10,*] Mean-Hwan Kim,[3,5,9] Dylan R. Muir,[3,6,9] M. Florencia Iacaruso,[3,7] Sonja B. Hofer,[2,3,8] and Thomas D. Mrsic-Flogel[2,3,8,*]

[1]Specification and Function of Neural Circuits Laboratory, The Francis Crick Institute, 1 Midland Road, London NW1 1AT, UK
[2]Sainsbury Wellcome Centre, 25 Howland Street, London W1T 4JG, UK
[3]Biozentrum, University of Basel, Klingelbergstrasse 70, 4056 Basel, Switzerland
[4]Present address: Specification and Function of Neural Circuits Laboratory, The Francis Crick Institute, 1 Midland Road, London NW1 1AT, UK
[5]Present address: Allen Institute for Brain Science, 615 Westlake Ave N, Seattle, WA 98109, USA
[6]Present address: SysSense, Thurgauerstrasse 60, 8050 Zürich, Switzerland
[7]Present address: Neuronal Circuits and Behaviour Laboratory, The Francis Crick Institute, 1 Midland Road, London NW1 1AT, UK
[8]Present address: Sainsbury Wellcome Centre, 25 Howland Street, London W1T 4JG, UK
[9]These authors contributed equally
[10]Lead contact
*Correspondence: petr.znamenskiy@crick.ac.uk (P.Z.), t.mrsic-flogel@ucl.ac.uk (T.D.M.-F.)
https://doi.org/10.1016/j.neuron.2023.12.013

SUMMARY

In the neocortex, neural activity is shaped by the interaction of excitatory and inhibitory neurons, defined by the organization of their synaptic connections. Although connections among excitatory pyramidal neurons are sparse and functionally tuned, inhibitory connectivity is thought to be dense and largely unstructured. By measuring *in vivo* visual responses and synaptic connectivity of parvalbumin-expressing (PV+) inhibitory cells in mouse primary visual cortex, we show that the synaptic weights of their connections to nearby pyramidal neurons are specifically tuned according to the similarity of the cells' responses. Individual PV+ cells strongly inhibit those pyramidal cells that provide them with strong excitation and share their visual selectivity. This structured organization of inhibitory synaptic weights provides a circuit mechanism for tuned inhibition onto pyramidal cells despite dense connectivity, stabilizing activity within feature-specific excitatory ensembles while supporting competition between them.

INTRODUCTION

Uncovering the synaptic organization of neural circuits is necessary for a mechanistic explanation of neural computation. Cortical excitatory neurons are organized into functional subnetworks, where nearby neurons with similar responses preferentially connect with strong synaptic connections.[1–5] However, how synaptic connectivity of inhibitory neurons and excitatory cells relates to their functional properties remains unresolved.[6–8] Reports of dense connectivity of parvalbumin-positive (PV+) neurons[6,7,9,10] have led to the view that these cells pool their inputs from the surrounding excitatory neurons and provide them with uniform inhibition[11–13] (Figure 1A), although some studies have challenged this conclusion.[8,14–17] However, while connectivity of PV+ neurons is dense, their synaptic connections have diverse efficacy and dynamics.[9,18–20] Several mutually exclusive connectivity motifs may account for this heterogeneity of synaptic strength (Figure 1A). At one extreme, PV+ neurons may preferentially inhibit cells with opposing functional properties.[21] At the other extreme, they may favor co-active cells providing feedback inhibition to the same population of pyramidal cells that drive them. Finally, they may distribute the strength of their connections randomly, providing uniform normalizing inhibition. Among these possibilities, feedback inhibition is consistent with the observation that inhibitory inputs onto individual pyramidal cells, while often broadly tuned, tend to match the tuning preference and strength of excitatory inputs,[22,23] even for features that are not organized in a columnar fashion.[24–26]

To disambiguate between these motifs, we characterized the organization of connection strength between PV+ and excitatory neurons and its relationship to their functional properties. We found that for reciprocally connected PV+/pyramidal neuron pairs, the strength of the inhibitory connections from the PV+ cell onto the pyramidal cell correlated with the strength of the excitatory connection from the pyramidal cell onto the PV+ cell. Furthermore, by relating *in vitro* connectivity of PV+/pyramidal neuron pairs to their activity *in vivo*, we show that although PV+ cells receive excitation from and inhibit the majority of nearby neurons, strong connections are found preferentially between cell pairs that are co-active during visual

 © 2023 The Author(s). Published by Elsevier Inc.
This is an open access article under the CC BY license (http://creativecommons.org/licenses/by/4.0/).

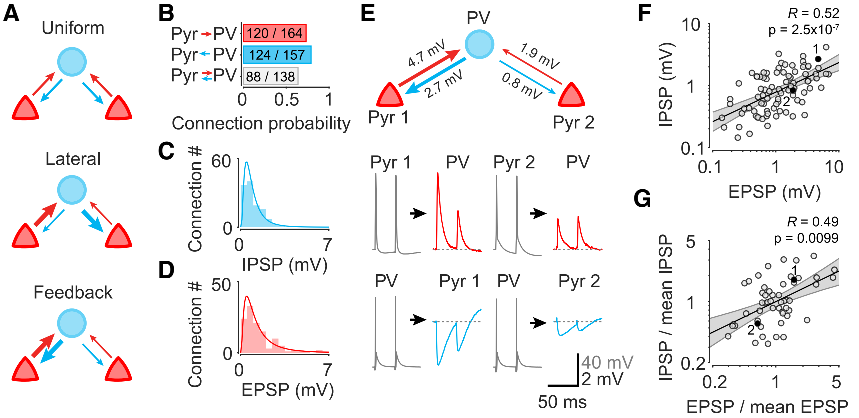

Figure 1. Heterogeneity of synaptic strength of PV+ neuron connections

(A) Three possible motifs of synaptic strength of PV+ neurons.

(B) Rates of pyramidal/PV+ neuron connectivity.

(C) Distribution of the strength of excitatory connections from pyramidal cells onto PV+ neurons.

(D) Distribution of the strength of inhibitory connections from PV+ neurons onto pyramidal cells.

(E) Example recording of a PV+ neuron reciprocally connected to two pyramidal cells. The PV+ neuron provides stronger inhibition to the pyramidal cell that provides it with stronger excitatory input.

(F) EPSP and IPSP strengths are correlated for reciprocally connected PV+/pyramidal neuron pairs. Black line: best fit regression line of log-IPSP magnitude against log-EPSP; gray shading: 95% confidence interval for the regression line estimated from bootstrap resampling; R and p are Pearson correlation and its p value, respectively. Cell pairs in (E) are highlighted.

(G) In recordings with multiple pyramidal neurons reciprocally connected to a PV+ cell, the correlation between EPSP and IPSP magnitude persists after controlling for slice quality by normalizing each by the geometric mean of EPSP/IPSP strength in the recording. Notation as in (F). p value was estimated using a shuffling procedure (see STAR Methods). See also Figures S1 and S2.

stimulation. Finally, using network simulations, we show that this pattern of tuned but broad inhibitory connectivity recapitulates several phenomena observed *in vivo*, including co-tuning of excitatory and inhibitory inputs received by individual pyramidal cells and competition between ensembles of differentially tuned pyramidal cells.

RESULTS

Individual PV+ neurons are strongly reciprocally connected with subpopulations of pyramidal cells

We examined the organization of connections of PV+ interneurons with pyramidal cells in layer 2/3 using whole-cell patch-clamp recordings in acute slices of primary visual cortex. We used transgenic mice expressing tdTomato in PV+ interneurons and confirmed the identity of putative pyramidal cells based on their regular spiking responses to current injection and excitatory input onto PV+ cells. Consistent with previous reports,[6,7,10,12] we found that PV+ cells were reciprocally connected with the majority of nearby excitatory neurons (Figure 1B). Among 138 cell pairs where we measured connectivity in both directions, we could detect post-synaptic potentials (PSPs) in both directions for 88 pairs (64%), only PV+ to Pyr inhibitory PSPs (IPSPs) for 21 pairs (15%), only Pyr to PV+ excitatory PSPs (EPSPs) for 12 pairs (9%), and no connections for 17 pairs (12%). However, the strengths of both excitatory inputs onto PV+ cells and their inhibitory inputs onto pyramidal cells spanned almost two orders of magnitude and followed a log-normal distribution (Figures 1C and 1D). We found no difference in the strength of connections between uni- and bi-directionally connected PV+-pyramidal neuron pairs (Figures S1D and S1E), in contrast with connections between pyramidal neurons.[1,27]

In reciprocally connected pairs, the magnitude of the EPSP onto the PV+ neuron was highly correlated with the magnitude of the IPSP onto the pyramidal cell (Figures 1E and 1F; 88 connections, including 58 PV+ cells and 79 pyramidal cells from 29 mice), as observed for reciprocal EPSPs between pyramidal neurons.[27] This relationship persisted when controlling for distance between patched cells (partial correlation $R = 0.57$, $p = 1.0 \times 10^{-8}$; Figures S1A–S1C) and could not be explained by variability in animal age between slice recordings (Figure S2). To exclude the possibility that the correlation between excitatory and inhibitory connection strength arose as a consequence of variability in slice quality, we examined recordings in which we simultaneously measured the strength of connections between a PV+ cell and multiple pyramidal cells and normalized the strength of excitatory and inhibitory connections by the geometric mean EPSP or IPSP magnitude of all simultaneously recorded cells. The correlation between the strength of inhibitory and excitatory connectivity persisted following this correction for variation in slice quality (Figures 1G and S1F; 53 pairs, including 23 PV+ cells from 16 mice; $p = 0.0099$, see STAR Methods). Together, these observations show that the strength of excitatory and inhibitory connections of PV+ interneurons is organized in a non-random manner. Specifically, individual PV+ cells preferentially provide strong feedback inhibition to the subset of nearby excitatory neurons from which they receive the strongest excitatory inputs.

PV+ neurons have diverse responses to visual stimuli

The modulation of reciprocal connection strength of PV+ cells with nearby pyramidal cells suggests that they primarily receive inputs from and provide inhibition to a subset of the local excitatory population. If this were the case, we might expect this heterogeneous connectivity to be reflected in the tuning properties of individual PV+ neurons, depending on which pyramidal cells provide them with strong excitatory drive. To test this, we recorded the visual responses of PV+ and PV− cells *in vivo* and then assayed the connectivity of the same cells in brain slices. We expressed GCaMP6f in transgenic mice expressing tdTomato in PV+ interneurons and recorded the visual responses of PV+ and nearby PV− cells in layer 2/3 of the primary visual cortex using two-photon calcium imaging (see STAR Methods). We used a rich stimulus set, including full-field gratings of varying direction and spatial and temporal frequency (Figures 2A and 2B; see STAR Methods).

CellPress
OPEN ACCESS

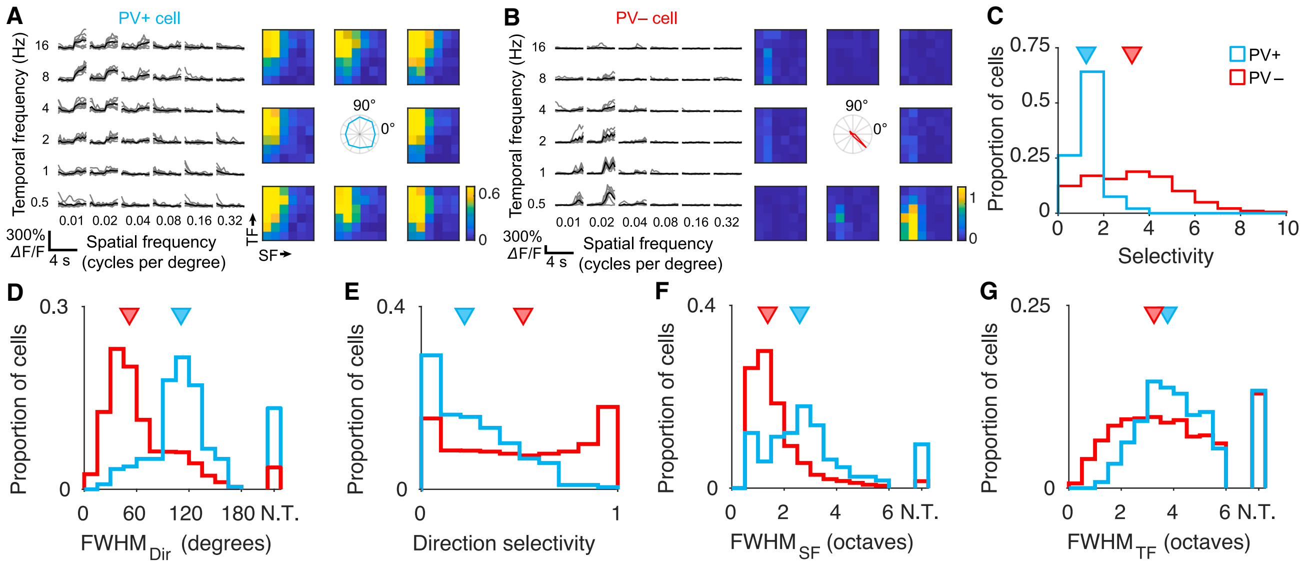

Figure 2. Response properties of PV+ neurons

(A and B) Visual responses of example PV+ (A) and PV− (B) neurons to drifting sinusoidal gratings. Traces show responses at the preferred direction (gray, single trials; black, mean response). Color maps show mean responses across spatial and temporal frequencies and directions during the moving phase of the grating.

(C) PV+ cells are less selective than PV− cells ($p = 1.2 \times 10^{-62}$, rank-sum test), quantified by skewness of their response distributions across stimulus types. Triangles indicate medians.

(D) PV+ cells are broadly tuned to orientation; FWHM, full width at half maximum response ($p = 2.3 \times 10^{-59}$, rank-sum test). N.T., cells untuned for orientation.

(E) PV+ cells are less direction selective than PV− cells ($p = 9.2 \times 10^{-28}$, rank-sum test).

(F and G) PV+ cells are more broadly tuned to spatial (C, $p = 7.7 \times 10^{-36}$, rank-sum test) and temporal (D, $p = 3.0 \times 10^{-8}$, rank-sum test) frequency than PV− cells.

We first quantified the tuning selectivity of single neurons by measuring the skewness of the distribution of responses over the entire stimulus set (288 stimuli). PV+ neurons tended to be less selective than pyramidal cells, responding to a broader range of stimuli (Figure 2C; $p = 1.6 \times 10^{-62}$, rank-sum test, 240 PV+ and 8,427 PV− neurons from 10 mice). In agreement with previous reports,[7,12,13] PV+ cells were broadly tuned to orientation and direction as well as spatial and temporal frequency (Figures 2D–2G).

However, responses of simultaneously recorded nearby PV+ cells were often functionally heterogeneous, responding to different combinations of directions and spatial and temporal frequencies (Figures 3A and 3B). To further characterize this functional heterogeneity, we computed the cosine similarity of trial-average responses (hence referred to as "response similarity") of PV− and PV+ cells. This metric quantifies how often PV+ and PV− cells responded to the same stimulus type. A cell pair with identical activity patterns will have a response similarity of 1, while two cells that are never active at the same time will have a response similarity of 0. Response similarity shows that individual PV+ cells have overlapping responses with different nearby PV− cells and thus are functionally affiliated with different excitatory subpopulations (Figure 3A). As a result, response similarity had a broad and positively skewed distribution, just like the distribution of response similarity of excitatory cell pairs[1] (Figure 3C).

Difference in response similarity between PV+/PV− cell pairs could not be trivially explained by preferences for individual stimulus features. For example, cells PV− #3 and PV+ #4 (Figure 3A) have similar directional tuning but low response similarity due to differences in their spatial and temporal frequency preferences. To quantify this, we next systematically examined the relationship between response similarity and feature selectivity as well as spatial distance of simultaneously recorded PV+/PV− cells pairs (Figures 3D–3G). Differences in preferred direction and spatial or temporal frequency were all weakly correlated with response similarity (Figure 3H), showing that, in isolation, they each explain only a small fraction of the variance in response similarity. There are two primary reasons behind these observations. First, as PV+ cells are broadly tuned, their preferred direction and spatial and temporal frequency are poor metrics for their overall stimulus response properties. Consequently, PV+ and PV− cell pairs can have a high response similarity if the preferred stimulus of the PV+ cell is different. Second, PV− cells tend to be highly selective and respond to a specific conjunction of direction and spatial and temporal frequency (Figure 2B). Therefore, response similarity can be low, even if stimulus preferences match along a subset of stimulus dimensions (as seen for PV− #3 and PV+ #4 in Figure 3A).

Previous work suggested that feature selectivity of PV+ neurons can in part be explained by local biases in tuning of pyramidal cells,[12,13] suggesting that PV+ neuron responses might be most similar to nearby PV− cells. To determine whether physical proximity could explain the range of response similarity values in our dataset, we examined response similarity as a function of distance between PV+/PV− cell pairs. Over the range of distances sampled in our dataset, however, distance had almost no effect on their response similarities (Figures 3G and 3H).

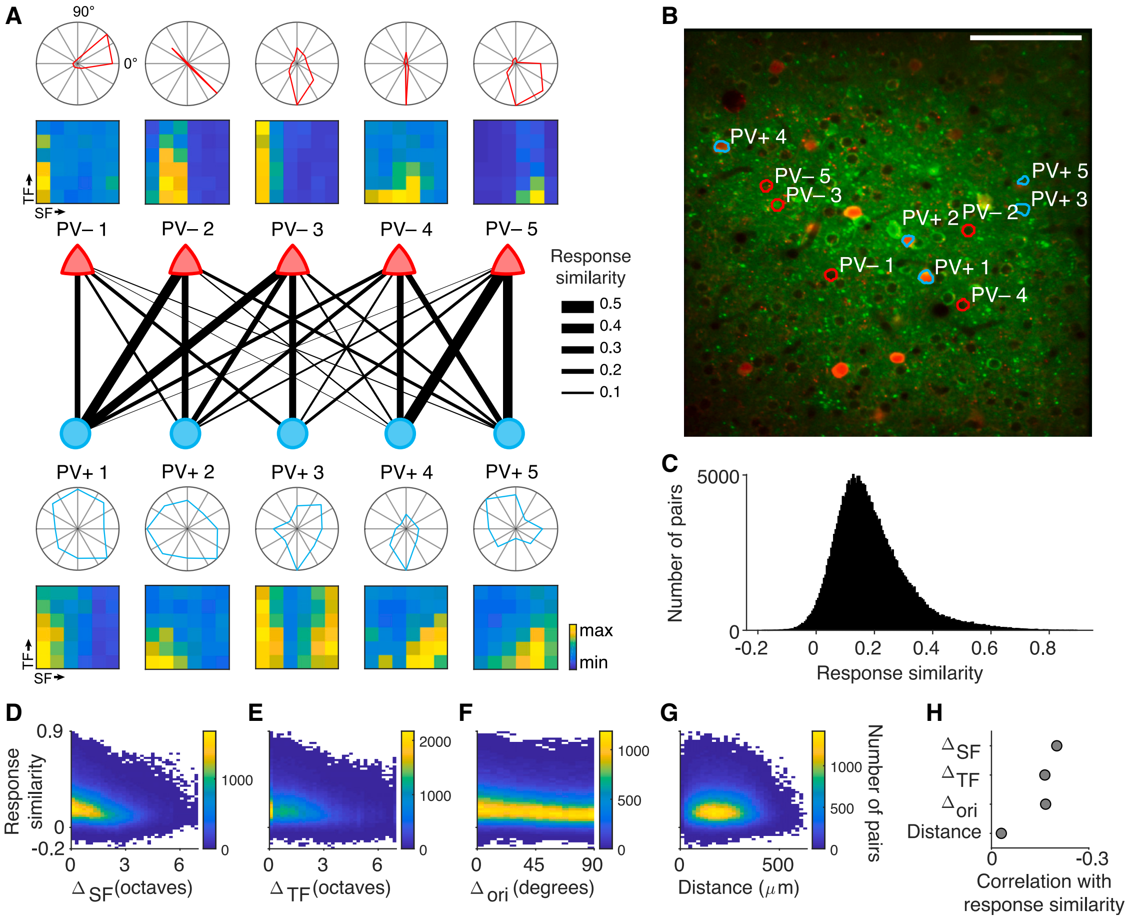

Figure 3. Response similarity of PV+/PV− neuron pairs is heterogeneous

(A) Tuning properties and response similarity of example simultaneously recorded PV+ and PV− neurons. Response similarities < 0 are not shown.

(B) Mean fluorescence image and spatial location of example neurons shown in (A). Note some neurons are located in other imaging planes. Scale bars, 100 μm.

(C) Distribution of response similarity of PV+/PV− neuron pairs (234,994 pairs between 255 PV+ and 8,427 PV− neurons).

(D–G) Two-dimensional histograms showing the relationship between response similarity and differences in preferred spatial frequency (D), temporal frequency (E), orientation (F), or distance between PV+/PV− cell pairs.

(H) Correlation coefficients of response similarity and differences in preferred spatial frequency, temporal frequency, orientation, or distance.

These results show that visual responses of nearby PV+ cells are heterogeneous and are poorly described by their selectivity for individual stimulus features in isolation. Instead, the overlap in tuning between PV+ and PV− neurons is better captured by response similarity, which takes into account their entire response profile.

Response similarity predicts the synaptic strength of PV+ neuron connections

Together, the observations above point to the possibility that the PV+ neurons are strongly and reciprocally connected to those nearby pyramidal cells that share their tuning for visual stimuli. To test this directly, we identified *in vitro* pyramidal and PV+ cell pairs whose visual responses we previously characterized *in vivo*[1,3,7] (Figures 4A–4C). We then examined the relationship between mean visual responses of PV+/pyramidal neuron pairs and their connection probabilities using the response similarity metric described above.

Consistent with previous studies of PV+ neuron connectivity,[6,7,10] we found that they provided inhibition to and received excitatory input from the majority of nearby excitatory cells, independent of the similarity of their tuning (Figures 4D and 4E; 67 PV+ to Pyr pairs from 22 mice, 64 Pyr to PV+ pairs from 23 mice). In contrast with connection probability, however, the magnitude of IPSPs from PV+ cells onto pyramidal cells was positively correlated with their response similarity measured *in vivo* (Figures 4F and S3A; 52 pairs from 18 mice, $R = 0.43$, $p = 0.0013$; Figure S4D, signal correlation). The strength of excitatory connections from pyramidal cells onto PV+ cells showed a similar correlation (Figures 4G, 5D, and S3B; 40 pairs from 19 mice, $R = 0.39$, $p = 0.013$). The similarity of single-trial responses of PV+ and pyramidal neurons ("total similarity"), a metric that takes into account trial-to-trial fluctuations in neuronal activity, showed a similar correlation with inhibitory connection strength but was not significantly correlated with excitatory connection strength (Figures 4H and S4–S6). As

CellPress
OPEN ACCESS

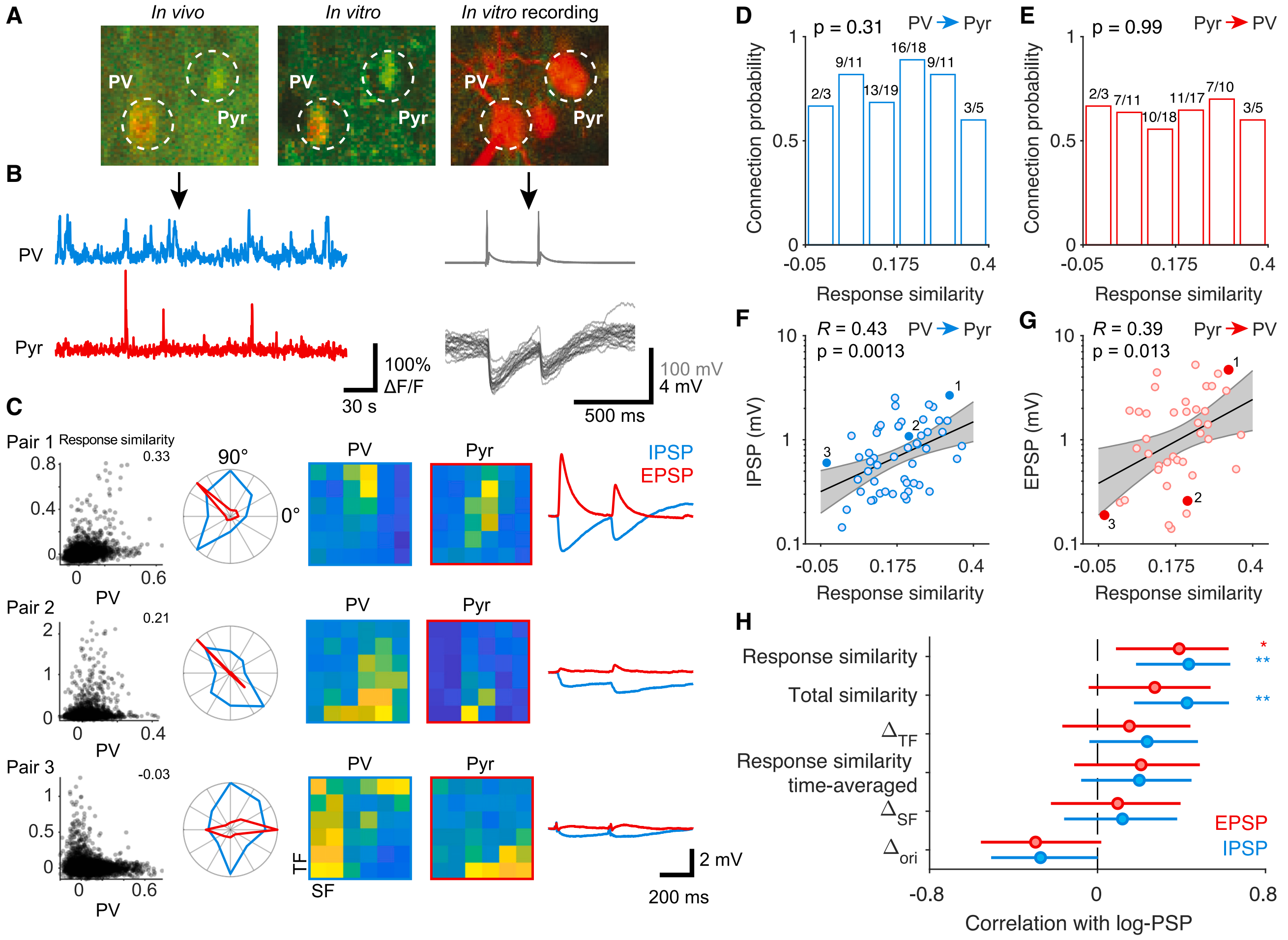

Figure 4. Response similarity predicts the strength of PV+ neuron connections

(A) Example PV-pyramidal neuron pair shown in vivo (left), in the brain slice (center), and during whole-cell recording (right). The *in vivo* image was generated by resampling the *in vivo* z stack to match to the coordinate frame of the in vitro volume.

(B) Example *in vivo* calcium traces (left) of the cells in (A) and *in vitro* current-clamp recordings of evoked action potentials in the PV+ cell (top right) and IPSPs in the pyramidal cell (bottom right).

(C) Left: scatterplots of dF/F trial-average responses on individual imaging frames across the stimulus ensemble for 3 reciprocally connected PV/pyramidal cell pairs. Center: visual tuning (blue, PV+ cell; red, pyramidal cell). Polar plots show normalized responses at spatial and temporal frequency that evoked the highest mean response. Colormaps show the normalized mean spatial and temporal frequency responses across directions. Right: post-synaptic potentials. Cell pair 1 is depicted in (A) and (B).

(D and E) Frequency of inhibitory connections from PV+ onto pyramidal cells (D) and excitatory connections from pyramidal cell onto PV+ cells (E) does not depend on response similarity. p value corresponds to the slope coefficient of logistic regression of connection probability against response similarity.

(F) Relationship of the strength of inhibitory connections from PV+ onto pyramidal cells and response similarity. Cell pairs shown in (C) are highlighted. Black line: best fit regression line of log-IPSP magnitude against response similarity; gray shading: 95% confidence interval for the regression line estimated by bootstrap resampling; R and p are Pearson correlation and its p value, respectively.

(G) Relationship of the strength of excitatory connections from pyramidal onto PV+ cells and response similarity. Notation as in (F).

(H) Similarity of trial-average responses but not selectivity for individual visual features predicts the strength of inhibitory connections from PV+ cells onto pyramidal cells (blue) and excitatory connections from pyramidal cells onto PV+ cells (red). Error bars are 95% confidence intervals. **$p < 0.01$, *$p < 0.05$. See also Figures S3–S7.

trial-average responses are as good or better at predicting PSP amplitude than single-trial responses, our data indicate that visually evoked rather than spontaneous activity governs the strength of input and output connections of PV+ neurons.

The correlation between response similarity and connection strength was not statistically significant if we discarded information about the time course of the cells' responses or examined the similarity of direction and spatial or temporal frequency tuning in isolation (Figures 4H and S4–S6). This is consistent with our observation that selectivity for individual stimulus features is only weakly correlated with overall response similarity of PV+/PV− neuron pairs (Figures 3D–3H).

Both inhibitory and excitatory post-synaptic responses varied in their short-term plasticity and decay dynamics (Figure S7). However, we found no correlation between response similarity and other synaptic properties, including paired pulse ratios

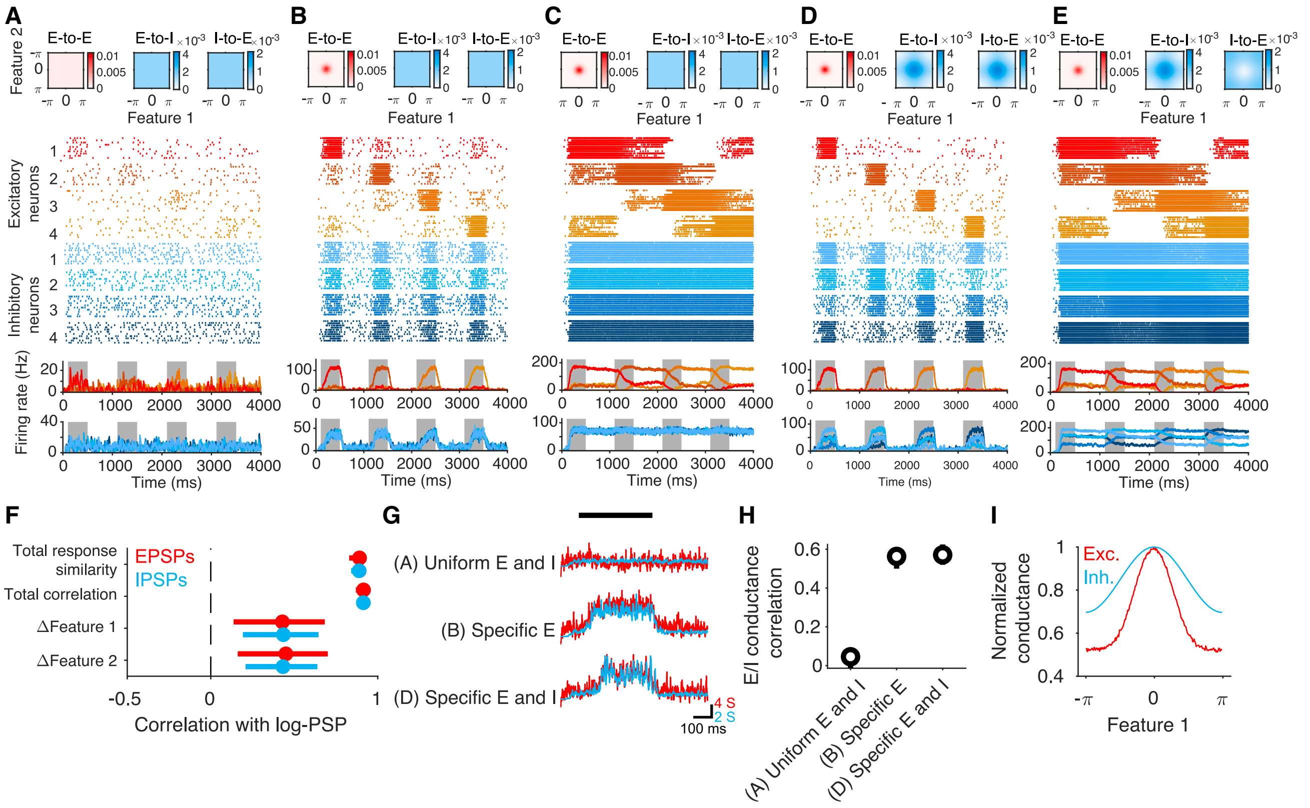

Figure 5. Simulations of networks with specific inhibitory connectivity demonstrate their role in network stability and recapitulate *in vivo* observations

(A–E) Networks with different distributions of excitatory and inhibitory synaptic strength. Top: connectivity matrices for E-to-E, E-to-I, and I-to-E connections; bottom: responses of example neurons to stimuli spanning feature 1 at the preferred value for the other stimulus dimension. (A) Network with uniform excitatory and inhibitory connectivity. Responses of example neurons (B) Specific excitatory connectivity between neurons co-tuned for both stimulus features ($s_E = 0.48$, see STAR Methods) enables amplification of neuronal responses. (C) Further increase in the specificity of excitatory connections ($s_E = 0.55$) leads to network instability. (D) Specific inhibitory connectivity ($s_I = 0.8$) between neurons co-tuned for both stimulus features balanced excitatory specificity, restoring network stability. (E) On the other hand, lateral inhibition, whereby inhibitory neurons strongly target excitatory neurons with different stimulus preferences ($s_I = -0.8$), promotes instability.

(F) In the network in (D), measures of overall response similarity are better predictors of connection strength of inhibitory neurons than individual feature preferences. Error bars, 95% confidence interval computed by subsampling connections using the sample sizes as in the experiments in Figure 4H.

(G and H) Excitatory and inhibitory inputs received by excitatory cells are correlated in networks with specific excitatory connections, independent of the presence of specific inhibitory connectivity.

(I) Inhibition received by excitatory cells in the network in (D) is co-tuned with excitation for individual stimulus features.

(Figures S7A and S7D) or response decay time constants (Figures S7B and S7E). Furthermore, the correlation between response similarity and the magnitude of post-synaptic responses persisted if we excluded pairs with slowly decaying responses from the analysis ($\tau_d > 25$ ms, Figures S7C and S7F). These analyses suggest that response similarity reflects the strength but not the dynamics of PV+ neuron connections, with stronger connections linking pairs of PV+/pyramidal cells with similar visual responses and weaker connections between cells that are rarely co-active.

Simulations of recurrent networks with specific inhibitory connectivity recapitulate *in vivo* observations

The computational role of inhibition has been studied in network models with structured or unstructured connectivity between excitatory and inhibitory neurons.[28–35] Our results indicate that the strength of connections of PV+ neurons with pyramidal cells in the primary visual cortex is tuned according to the overall similarity of their visual responses. To specifically explore the impact of such connectivity motifs on circuit function, we built a spiking recurrent network model wherein connection strength was determined by the conjunction of tuning for two stimulus features (e.g., direction and spatial frequency). We varied the degree of *specificity* of excitatory and inhibitory connections in the network, a parameter that defines the proportion of total synaptic strength, assigned according to the similarity of their stimulus preferences, with the remainder of synapses distributed uniformly across the network (see STAR Methods).

We first consider a network with uniform excitatory and inhibitory connections (excitatory and inhibitory specificity parameters set to zero; Figure 5A). Feedforward inputs tuned for both stimulus features resulted in weak and unreliable responses in

CellPress
OPEN ACCESS

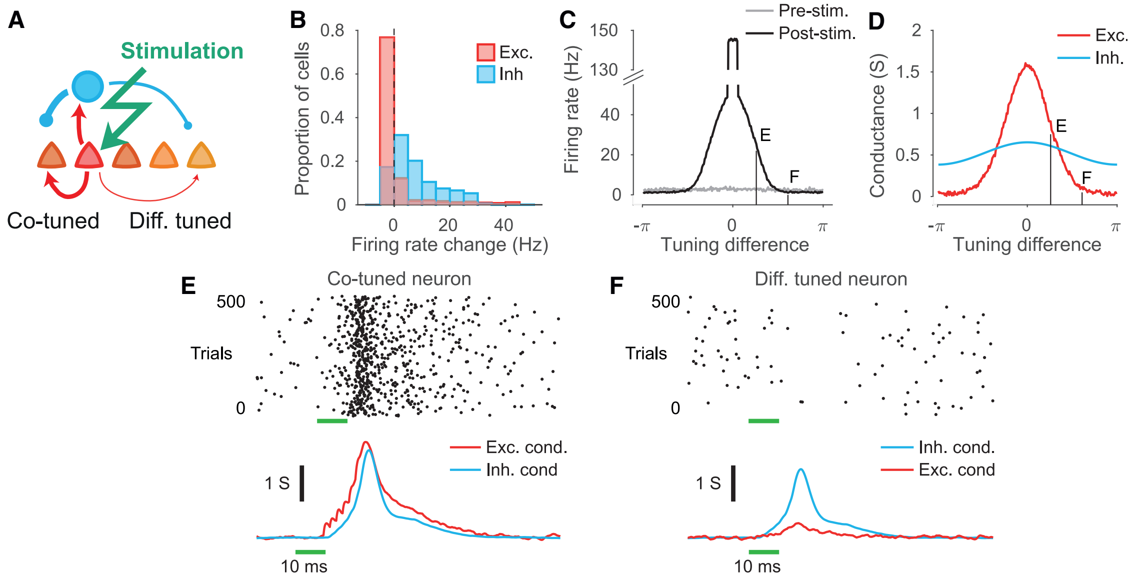

Figure 6. Specific but broad inhibitory connectivity supports neuronal competition

(A and B) Stimulation of a small cohort of excitatory neurons (11 of 4,000) in the network shown in Figure 5C leads to facilitation of the majority of inhibitory neurons and suppression of the majority of excitatory neurons (B).

(C–F) In the subset of excitatory neurons with similar tuning to the stimulated population, stimulation boosts activity (C and E) driven by excitatory inputs exceeding inhibition (D). In other cells, stimulation suppresses firing (C and F) driven by strong multi-synaptic inhibitory inputs in the absence of excitatory inputs (D).

excitatory neurons, while inhibitory neurons were unselective (Figure 5A). Next, we increased the specificity of excitatory connections, assigning a greater proportion of recurrent excitatory connections to co-tuned neurons. Increasing specificity of excitatory connections enabled the network to amplify sensory inputs (Figure 5B). However, specific excitatory connectivity eventually led to network instability, whereby activity persisted in the absence of external input driven by recurrent excitation (Figure 5C). Consequently, tuned inputs drove excitatory neurons to fire at saturation and their firing did not seize until the next stimulus presentation. Next, we adjusted the distribution of inhibitory connection strength according to the conjunction of neurons' tuning preferences (Figure 5D), resembling the connectivity observed experimentally (Figures 4F–4H). This network regained stability and responded robustly but transiently to tuned inputs, similar to *in vivo* measurements of neural activity in sensory areas of the neocortex. Finally, we examined the consequences of introducing negative inhibitory specificity, whereby inhibitory connections are strongest between inhibitory and excitatory cells tuned to different stimuli, in the network, with specific excitatory connections depicted in Figure 5B. This redistribution of inhibitory connection strength also pushed the network into an unstable regime (Figure 5E). These simulations demonstrate that the excitatory and inhibitory connectivity rules play opposing roles in controlling the stability of recurrent networks.

The behavior of the network with specific excitatory and inhibitory connections depicted in Figure 5D is consistent with a broad array of existing experimental findings. First, the strength of connections between inhibitory and excitatory neurons is best predicted by the overall similarity of their firing patterns rather than the similarity of their preference for individual stimulus features (Figure 5F), consistent with our experimental observations (Figure 4H). This illustrates that, when preferences for multiple stimulus features jointly constrain the strength of synaptic connections, the correlation between synaptic weights and similarity of tuning for individual stimulus features can be difficult to detect. Next, excitatory and inhibitory inputs onto individual pyramidal cells were correlated on fine timescales (Figures 5G and 5H).[36] This correlation was also observed in the network, with specific excitatory connections but with uniform inhibitory connectivity. Finally, total inhibitory input received by individual pyramidal cells is broad but co-tuned with excitatory inputs for individual stimulus features (Figure 5I), in agreement with *in vivo* intracellular measurements of excitatory and inhibitory currents in pyramidal neurons in the V1.[25]

Computational models in cortical networks emphasize the role of global or counter-tuned inhibitory feedback in neuronal competition and selectivity.[21,28,30,33,34,37–39] Strong inhibition onto co-tuned neurons, as we found in the mouse V1, is seemingly ill-suited to mediate competition between subpopulations of neurons with distinct response tuning. To explore the role of specific co-tuned inhibition in neuronal competition, we examined the network effects of stimulating a small cohort of co-tuned neurons in the network with specific excitatory and inhibitory connections (Figure 6A; see STAR Methods). This manipulation resulted in an increase in firing in the majority of inhibitory neurons within the network and a suppression of firing in the majority of excitatory neurons, while strongly activating a small subset of them (Figure 6B). Specifically, stimulation increased the firing rate of cells whose tuning was similar to the activated cohort for *both* stimulus features (Figure 6C). Although stimulation

triggered the strongest inhibitory input to this subpopulation, they were offset by even greater recurrent excitation (Figures 6D and 6E). On the other hand, inhibition dominated in those neurons with tuning distinct from the stimulated cohort, resulting in suppression of firing (Figures 6D and 6F). Importantly, stimulation inhibited neurons that matched the stimulated cohort for one stimulus feature but had different tuning for the other (Figure 6C). Thus, specific inhibitory and excitatory connectivity can act in concert to facilitate stable amplification within subpopulations of cortical neurons co-tuned along multiple stimulus dimensions, while promoting competition between subpopulations that differ in stimulus preferences.

DISCUSSION

Our results show that the strength of connections between PV+ interneurons and pyramidal cells in the visual cortex follows a similar rule to that of recurrent excitatory connectivity[1]: inhibitory neurons receive stronger excitation from, and provide stronger inhibition to, a subset of nearby excitatory neurons, specifically those with similar responses to visual stimulation. Although the correlations between the strength of synaptic connections and response similarity were significant, they accounted for a minority of variance in synaptic strength (18% for IPSPs and 15% for EPSPs). The remaining variance may reflect both biological and technical sources, such as variation in slice quality. The grating stimuli used in our experiments did not directly probe the receptive field structure of PV+ and PV− neurons. As our results show that the strength of PV+ neuron connections relates to the overall pattern of their visual responses, it is likely that receptive field structure also contributes to the organization of their synaptic strength. Taking into account receptive field structure may help further explain the variability in the strength of PV+ neuron connections.

This like-to-like connectivity constrains models of recurrent networks with structured synaptic connections[29,31,32] and provides a unifying explanation for a range of experimental observations. Despite the sea of dense connections between inhibitory and excitatory cells,[6,7,10,15,40] the specificity of excitatory synaptic weights onto PV+ neurons may contribute to the diversity of their preferences for visual features.[7,13,16,41] At the same time, the specificity of inhibitory connection strength facilitates fine-tuning of inhibition received by individual excitatory neurons and may give rise to the correlations between inhibitory and excitatory synaptic inputs observed in intracellular recordings.[22,24–26,36,42] Functionally specific inhibitory connections may be the circuit implementation of network models reliant on co-tuned feedback inhibition[35,43–45] and serve to stabilize the dynamics of strongly connected, recurrent subnetworks of co-active excitatory neurons.[1]

Experiments using single-cell stimulation have suggested that exciting single V1 neurons typically results in the facilitation of activity of cells with highly similar responses and suppression of less similarly tuned cells.[46] These effects can be explained as the result of the interactions between the specific inhibitory connectivity we describe here with even more specific excitatory connections.[1,3] This interpretation is further supported by recent experiments stimulating ensembles of excitatory V1 neurons, which could only be accurately explained by models that incorporate like-to-like connectivity for both excitatory and inhibitory inputs,[47] as we describe above.

Our network simulations illustrate the difficulty of detecting recurrent amplification in such experiments because this requires knowledge of all tuning dimensions that govern their synaptic connectivity. For example, pyramidal neurons that prefer grating stimuli with similar direction and spatial and temporal frequency, but respond to opposite phases of the grating, will rarely fire together and are expected to excite each other only rarely and with weak connections,[1] while inhibiting each other via weak but dense disynaptic inhibition. Consequently, we should expect that stimulation of one of these cells will result in the net inhibition of firing of the other (Figure 6).

Recent work using electron microscopy to reconstruct inhibitory connectivity suggests that inhibitory neurons in the mouse posterior parietal cortex provide opponent inhibition to excitatory neurons with the opposite selectivity.[14] However, this effect was not observed for basket cells, which constitute the majority of PV+ neurons. Together with our results, this observation suggests that the connectivity of different classes of inhibitory neurons may follow different organizing principles.

Our findings raise the question of how the organization of the excitatory and inhibitory connection strength of PV+ neurons is established. On one hand, both excitatory and inhibitory connections may be shaped by activity-dependent wiring rules driven by the patterns of correlated firing in the network.[48–50] Alternatively, in the presence of heterogeneous but random connectivity from pyramidal cells onto inhibitory neurons, homeostatic plasticity of inhibitory connections alone may be sufficient, as suggested by recent modeling work.[51] Because synaptic weights of interneurons and pyramidal cells are determined by their overall response similarity rather than selectivity for individual features of visual stimuli, we speculate that this wiring rule may be shared by other regions of the cortex.

STAR★METHODS

Detailed methods are provided in the online version of this paper and include the following:

- KEY RESOURCES TABLE
- RESOURCE AVAILABILITY
  - Lead contact
  - Materials availability
  - Data and code availability
- EXPERIMENTAL MODEL AND STUDY PARTICIPANT DETAILS
- METHOD DETAILS
  - Responses and connectivity of PV interneurons
  - Models of specific inhibitory and excitatory connectivity
- QUANTIFICATION AND STATISTICAL ANALYSIS
  - Data analysis: preprocessing
  - Data analysis: responsiveness and selectivity
  - Data analysis: direction and SF/TF tuning
  - Data analysis: synaptic connectivity
  - Data analysis: relating synaptic connectivity and *in vivo* responses

CellPress
OPEN ACCESS

SUPPLEMENTAL INFORMATION

Supplemental information can be found online at https://doi.org/10.1016/j.neuron.2023.12.013.

ACKNOWLEDGMENTS

This work was supported by the Francis Crick Institute, which receives its core funding from Cancer Research UK (CC2108), the UK Medical Research Council (CC2108), and the Wellcome Trust (CC2108); by the Sainsbury Wellcome Centre, which receives funding from the Wellcome Trust (219627/Z/19/Z) and the Gatsby Charitable Foundation (GAT3755); and by grants from the European Commission (PIIF-GA-2013-625740 to P.Z.), the European Research Council (NeuroV1sion 616509 to T.D.M.-F.), and the Swiss National Science Foundation (SNSF 31003A_169802 to T.D.M.-F.). For the purpose of Open Access, the author has applied a CC BY public copyright license to any Author Accepted Manuscript version arising from this submission. We thank M. Rio for the development of the imaging pre-processing pipeline, M. Li for animal husbandry, and L. Cossell for comments on the manuscript.

AUTHOR CONTRIBUTIONS

S.B.H., T.D.M.-F., P.Z., M.-H.K., and M.F.I. designed the experiments; P.Z., M.-H.K., and M.F.I. carried out the experiments; P.Z., M.-H.K., and T.D.M.-F. analyzed the data; D.R.M. and P.Z. performed the network simulations; P.Z., D.R.M., and T.D.M.-F. wrote the paper.

DECLARATION OF INTERESTS

The authors declare no competing interests.

Received: June 15, 2023
Revised: October 31, 2023
Accepted: December 19, 2023
Published: January 19, 2024

REFERENCES

1. Cossell, L., Iacaruso, M.F., Muir, D.R., Houlton, R., Sader, E.N., Ko, H., Hofer, S.B., and Mrsic-Flogel, T.D. (2015). Functional organization of excitatory synaptic strength in primary visual cortex. Nature *518*, 399–403.
2. Ding, Z., Fahey, P.G., Papadopoulos, S., Wang, E.Y., Celii, B., Papadopoulos, C., Kunin, A.B., Chang, A., Fu, J., Ding, Z., et al. (2023). Functional connectomics reveals general wiring rule in mouse visual cortex. Preprint at bioRxiv. https://doi.org/10.1101/2023.03.13.531369.
3. Ko, H., Hofer, S.B., Pichler, B., Buchanan, K.A., Sjöström, P.J., and Mrsic-Flogel, T.D. (2011). Functional specificity of local synaptic connections in neocortical networks. Nature *473*, 87–91.
4. Morgenstern, N.A., Bourg, J., and Petreanu, L. (2016). Multilaminar networks of cortical neurons integrate common inputs from sensory thalamus. Nat. Neurosci. *19*, 1034–1040.
5. Yoshimura, Y., Dantzker, J.L.M., and Callaway, E.M. (2005). Excitatory cortical neurons form fine-scale functional networks. Nature *433*, 868–873.
6. Bock, D.D., Lee, W.C., Kerlin, A.M., Andermann, M.L., Hood, G., Wetzel, A.W., Yurgenson, S., Soucy, E.R., Kim, H.S., and Reid, R.C. (2011). Network anatomy and in vivo physiology of visual cortical neurons. Nature *471*, 177–182.
7. Hofer, S.B., Ko, H., Pichler, B., Vogelstein, J., Ros, H., Zeng, H., Lein, E., Lesica, N.A., and Mrsic-Flogel, T.D. (2011). Differential connectivity and response dynamics of excitatory and inhibitory neurons in visual cortex. Nat. Neurosci. *14*, 1045–1052.
8. Rossi, L.F., Harris, K.D., and Carandini, M. (2020). Spatial connectivity matches direction selectivity in visual cortex. Nature *588*, 648–652.
9. Holmgren, C., Harkany, T., Svennenfors, B., and Zilberter, Y. (2003). Pyramidal cell communication within local networks in layer 2/3 of rat neocortex. J. Physiol. *551*, 139–153.
10. Packer, A.M., and Yuste, R. (2011). Dense, unspecific connectivity of neocortical parvalbumin-positive interneurons: a canonical microcircuit for inhibition? J. Neurosci. *31*, 13260–13271.
11. Harris, K.D., and Mrsic-Flogel, T.D. (2013). Cortical connectivity and sensory coding. Nature *503*, 51–58.
12. Kerlin, A.M., Andermann, M.L., Berezovskii, V.K., and Reid, R.C. (2010). Broadly tuned response properties of diverse inhibitory neuron subtypes in mouse visual cortex. Neuron *67*, 858–871.
13. Scholl, B., Pattadkal, J.J., Dilly, G.A., Priebe, N.J., and Zemelman, B.V. (2015). Local integration accounts for weak selectivity of mouse neocortical parvalbumin interneurons. Neuron *87*, 424–436.
14. Kuan, A.T., Bondanelli, G., Driscoll, L.N., Han, J., Kim, M., Hildebrand, D.G.C., Graham, B.J., Thomas, L.A., Panzeri, S., Harvey, C.D., et al. (2022). Synaptic wiring motifs in posterior parietal cortex support decision-making. Preprint at bioRxiv. https://doi.org/10.1101/2022.04.13.488176.
15. Runyan, C.A., Schummers, J., Van Wart, A., Kuhlman, S.J., Wilson, N.R., Huang, Z.J., and Sur, M. (2010). Response features of parvalbumin-expressing interneurons suggest precise roles for subtypes of inhibition in visual cortex. Neuron *67*, 847–857.
16. Wilson, D.E., Smith, G.B., Jacob, A.L., Walker, T., Dimidschstein, J., Fishell, G., and Fitzpatrick, D. (2017). GABAergic neurons in ferret visual cortex participate in functionally specific networks. Neuron *93*, 1058–1065.e4.
17. Yoshimura, Y., and Callaway, E.M. (2005). Fine-scale specificity of cortical networks depends on inhibitory cell type and connectivity. Nat. Neurosci. *8*, 1552–1559.
18. Buhl, E.H., Halasy, K., and Somogyi, P. (1994). Diverse sources of hippocampal unitary inhibitory postsynaptic potentials and the number of synaptic release sites. Nature *368*, 823–828.
19. D'Souza, R.D., Bista, P., Meier, A.M., Ji, W., and Burkhalter, A. (2019). Spatial clustering of inhibition in mouse primary visual cortex. Neuron *104*, 588–600.e5.
20. Strüber, M., Jonas, P., and Bartos, M. (2015). Strength and duration of perisomatic GABAergic inhibition depend on distance between synaptically connected cells. Proc. Natl. Acad. Sci. USA *112*, 1220–1225.
21. Troyer, T.W., Krukowski, A.E., Priebe, N.J., and Miller, K.D. (1998). Contrast-invariant orientation tuning in cat visual cortex: thalamocortical input tuning and correlation-based intracortical connectivity. J. Neurosci. *18*, 5908–5927.
22. Wehr, M., and Zador, A.M. (2003). Balanced inhibition underlies tuning and sharpens spike timing in auditory cortex. Nature *426*, 442–446.
23. Xue, M., Atallah, B.V., and Scanziani, M. (2014). Equalizing excitation–inhibition ratios across visual cortical neurons. Nature *511*, 596–600.
24. Li, Y.T., Ma, W.P., Li, L.Y., Ibrahim, L.A., Wang, S.Z., and Tao, H.W. (2012). Broadening of inhibitory tuning underlies contrast-dependent sharpening of orientation selectivity in mouse visual cortex. J. Neurosci. *32*, 16466–16477.
25. Liu, B.H., Li, Y.T., Ma, W.P., Pan, C.J., Zhang, L.I., and Tao, H.W. (2011). Broad inhibition sharpens orientation selectivity by expanding input dynamic range in mouse simple cells. Neuron *71*, 542–554.
26. Tan, A.Y.Y., Brown, B.D., Scholl, B., Mohanty, D., and Priebe, N.J. (2011). Orientation selectivity of synaptic input to neurons in mouse and cat primary visual cortex. J. Neurosci. *31*, 12339–12350.
27. Song, S., Sjöström, P.J., Reigl, M., Nelson, S., and Chklovskii, D.B. (2005). Highly nonrandom features of synaptic connectivity in local cortical circuits. PLoS Biol. *3*, e68.
28. Ben-Yishai, R., Bar-Or, R.L., and Sompolinsky, H. (1995). Theory of orientation tuning in visual cortex. Proc. Natl. Acad. Sci. USA *92*, 3844–3848.

29. Doiron, B., and Litwin-Kumar, A. (2014). Balanced neural architecture and the idling brain. Front. Comput. Neurosci. *8*, 56.
30. Hansel, D., and van Vreeswijk, C. (2012). The mechanism of orientation selectivity in primary visual cortex without a functional map. J. Neurosci. *32*, 4049–4064.
31. Litwin-Kumar, A., Rosenbaum, R., and Doiron, B. (2016). Inhibitory stabilization and visual coding in cortical circuits with multiple interneuron subtypes. J. Neurophysiol. *115*, 1399–1409.
32. Litwin-Kumar, A., and Doiron, B. (2012). Slow dynamics and high variability in balanced cortical networks with clustered connections. Nat. Neurosci. *15*, 1498–1505.
33. Pehlevan, C., and Sompolinsky, H. (2014). Selectivity and sparseness in randomly connected balanced networks. PLoS One *9*, e89992.
34. Sadeh, S., Cardanobile, S., and Rotter, S. (2014). Mean-field analysis of orientation selectivity in inhibition-dominated networks of spiking neurons. SpringerPlus *3*, 148.
35. Somers, D.C., Nelson, S.B., and Sur, M. (1995). An emergent model of orientation selectivity in cat visual cortical simple cells. J. Neurosci. *15*, 5448–5465.
36. Okun, M., and Lampl, I. (2008). Instantaneous correlation of excitation and inhibition during ongoing and sensory-evoked activities. Nat. Neurosci. *11*, 535–537.
37. Ernst, U., Pawelzik, K., Tsodyks, M., and Sejnowski, T.J. (1999). Relation between retinotopical and orientation maps in visual cortex. Neural Comput. *11*, 375–379.
38. Li, Z. (1998). A neural model of contour integration in the primary visual cortex. Neural Comput. *10*, 903–940.
39. Shushruth, S., Mangapathy, P., Ichida, J.M., Bressloff, P.C., Schwabe, L., and Angelucci, A. (2012). Strong recurrent networks compute the orientation tuning of surround modulation in the primate primary visual cortex. J. Neurosci. *32*, 308–321.
40. Fino, E., and Yuste, R. (2011). Dense inhibitory connectivity in neocortex. Neuron *69*, 1188–1203.
41. Keller, A.J., and Martin, K.A.C. (2015). Local circuits for contrast normalization and adaptation investigated with two-photon imaging in cat primary visual cortex. J. Neurosci. *35*, 10078–10087.
42. Borg-Graham, L.J., Monier, C., and Frégnac, Y. (1998). Visual input evokes transient and strong shunting inhibition in visual cortical neurons. Nature *393*, 369–373.
43. Denève, S., and Machens, C.K. (2016). Efficient codes and balanced networks. Nat. Neurosci. *19*, 375–382.
44. Murphy, B.K., and Miller, K.D. (2009). Balanced amplification: a new mechanism of selective amplification of neural activity patterns. Neuron *61*, 635–648.
45. Rubin, D.B., Van Hooser, S.D., and Miller, K.D. (2015). The stabilized supralinear network: a unifying circuit motif underlying multi-input integration in sensory cortex. Neuron *85*, 402–417.
46. Chettih, S.N., and Harvey, C.D. (2019). Single-neuron perturbations reveal feature-specific competition in V1. Nature *567*, 334–340.
47. Oldenburg, I.A., Hendricks, W.D., Handy, G., Shamardani, K., Bounds, H.A., Doiron, B., and Adesnik, H. (2022). The logic of recurrent circuits in the primary visual cortex. Preprint at bioRxiv. https://doi.org/10.1101/2022.09.20.508739.
48. Mackwood, O., Naumann, L.B., and Sprekeler, H. (2021). Learning excitatory-inhibitory neuronal assemblies in recurrent networks. eLife *10*, e59715.
49. Sadeh, S., Clopath, C., and Rotter, S. (2015). Emergence of functional specificity in balanced networks with synaptic plasticity. PLoS Comput. Biol. *11*, e1004307.
50. Vogels, T.P., Sprekeler, H., Zenke, F., Clopath, C., and Gerstner, W. (2011). Inhibitory plasticity balances excitation and inhibition in sensory pathways and memory networks. Science *334*, 1569–1573.
51. Lagzi, F., and Fairhall, A. (2022). Tuned inhibitory firing rate and connection weights as emergent network properties. Preprint at bioRxiv. https://doi.org/10.1101/2022.04.12.488114.
52. Orsolic, I., Rio, M., Mrsic-Flogel, T.D., and Znamenskiy, P. (2021). Mesoscale cortical dynamics reflect the interaction of sensory evidence and temporal expectation during perceptual decision-making. Neuron *109*, 1861–1875.e10.
53. Plesser, H.E., Diesmann, M., Gewaltig, M.-O., and Morrison, A. (2013). NEST: the neural simulation tool. In Encyclopedia of Computational Neuroscience, D. Jaeger. and R. Jung., eds. (Springer), pp. 1–4.
54. Kim, M.H., Znamenskiy, P., Iacaruso, M.F., and Mrsic-Flogel, T.D. (2018). Segregated subnetworks of intracortical projection neurons in primary visual cortex. Neuron *100*, 1313–1321.e6.

CellPress
OPEN ACCESS

STAR★METHODS

KEY RESOURCES TABLE

REAGENT or RESOURCE	SOURCE	IDENTIFIER
Bacterial and virus strains		
AAV1 hSyn-GCaMP6f	University of Pennsylvania Vector Core	N/A
Deposited data		
Preprocessed calcium imaging and in vitro connectivity data	This paper	Figshare: https://doi.org/10.25418/crick.23295188
Experimental models: Organisms/strains		
Mouse: Ai9 B6.Cg-Gt(ROSA) 26Sortm9(CAG-tdTomato)Hze/J	The Jackson Laboratory	RRID:IMSR_JAX:007909
Mouse: Ai14 B6.Cg-Gt(ROSA) 26Sortm14(CAG-tdTomato)Hze/J	The Jackson Laboratory	RRID:IMSR_JAX:007914
Mouse: PV-Cre B6;129P2-Pvalbtm1(cre)Arbr/J	The Jackson Laboratory	RRID:IMSR_JAX:008069
Software and algorithms		
GUI for control point registration of 3D image stacks	This paper	Zenodo: https://doi.org/10.5281/zenodo.10404664
Custom analysis code	This paper	Figshare: https://doi.org/10.25418/crick.23295188
ASt model for neuropil correction	Orsolic et al.[52]	Zenodo: https://doi.org/10.5281/zenodo.10404603
NEST Simulator	Plesser et al.[53]	RRID:SCR_002963

RESOURCE AVAILABILITY

Lead contact

Further information and requests for resources and reagents should be directed to Petr Znamenskiy (petr.znamenskiy@crick.ac.uk).

Materials availability

This study did not generate new unique reagents.

Data and code availability

- Preprocessed data, including *in vivo* fluorescence traces and *in vitro* recordings, have been deposited on Figshare. The DOI is listed in the key resources table. Raw image data is available from the lead contact upon request.
- Original code required to reproduce the analyses in this paper has been deposited on Figshare. The DOI is listed in the key resources table.
- Any additional information required to reanalyze the data reported in this work paper is available from the lead contact upon request.

EXPERIMENTAL MODEL AND STUDY PARTICIPANT DETAILS

All experiments were conducted in accordance with institutional animal welfare guidelines and licensed by the Swiss cantonal veterinary office. To label parvalbumin expressing interneurons, Ai9 (RRID:IMSR_JAX:007909) or Ai14 (RRID:IMSR_JAX:007914) LSL-tdTomato mice were crossed with PV-Cre mice (RRID:IMSR_JAX:008069). We used both male and female animals (P18–30 at the start of experiments).

METHOD DETAILS

Responses and connectivity of PV interneurons

Animals and surgical procedures

Animals were anaesthetized with 5 mg/kg midazolam, medetomidine 0.5 mg/kg, and 0.05 mg/kg fentanyl. A metal headplate was implanted exposing the skull over the right visual cortex and 60 nl of AAV 2.1 hSyn-GCaMP6f were injected into V1. To facilitate

identification of the injection site in vitro, the injection capillary was coated with DiI. Approximately 5 days after the injection, animals were anaesthetized again and a craniotomy 4 mm in diameter was made exposing the visual cortex. A glass coverslip (4 mm diameter, 0.17 mm thickness) was implanted for chronic calcium imaging.

In vivo *two-photon calcium imaging*

Awake mice were head-fixed and allowed to run on a styrofoam wheel. A monitor (47 cm wide) was placed 20–25 cm away from the eye spanning a field of view of 122 visual degrees. Monitor position was adjusted such that the center of the screen matched the preferred retinotopic location of the imaging site, as judged by two-photon fluorescence responses to grating patches flashed at different locations on the screen.

Fluorescence signals were recorded using a ThorLabs Bergamo and a ThorLabs Bergamo II resonant scanning two-photon microscopes with a Nikon 16x water-immersion objective (NA 0.8) operated using ScanImage 4 or 5.1 software. GCaMP6f fluorescence was imaged using 930 nm excitation at 10–30 mW with a 520/40 nm emission filter (Chroma). Volumes of 8 frames spanning 80 μm in depth and $\sim$350–520 μm in X-Y were acquired at 3.6-3.7 Hz using a piezo focuser (PI P-726). For identification of PV-positive neurons, tdTomato fluorescence was imaged at 930 nm with a 607/70 nm emission filter (Semrock).

To prevent light from the monitor from interfering with imaging, the monitor backlight was controlled by a custom electronic circuit and only switched on during the turn-around of the resonant X mirror. Sinusoidal gratings of 6 spatial frequencies, 6 temporal frequencies and 8 directions were interleaved randomly and presented without gaps. Each grating first remained stationary for 1.3 or 2.1 seconds (5 or 8 volumes), before moving for 2.1 seconds (8 volumes). A single presentation of the stimulus set constituted a single imaging segment, 6–10 of which were repeated during each imaging session.

In vivo/in vitro *registration*

Following the *in vivo* imaging session, animals were anaesthetized using isofluorane (1.5–2%). A detailed Z-stack of GCaMP6f, tdTomato, and DiI fluorescence at the imaging site was acquired at 830 nm, spanning the volume from 0 to $\sim$300 μm below the pia. Since GCaMP6f fluorescence at this wavelength does not depend on calcium concentration, neurons could be readily identified independent of their level of activity.

The slice containing the *in vivo* imaging site was identified *in vitro*, using DiI fluorescence and parenchymal blood vessels as landmarks. As *in vivo*, a Z-stack was acquired at 830 nm spanning all or most of the slice thickness. *In vivo* and *in vitro* volumes were then aligned using custom software written in Matlab (https://github.com/znamensk/RegisterStack). Four or more manually selected control points were used to estimate forward (*in vivo* to *in vitro*) and inverse (*in vitro* to *in vivo*) affine transformation matrices using least squares regression. The quality of the registration was verified by rotating and reslicing the *in vivo* Z-stack in the coordinate frame of the *in vitro* volume using the forward transformation. If necessary, control points were added or replaced until the registration quality was satisfactory. A similar procedure was used to register the *in vivo* Z-stack with the *in vivo* functional imaging planes.

The inverse transformation matrices were used to identify the *in vivo* imaging ROI corresponding to each of the neurons recorded *in vitro*. Registration accuracy was confirmed by manual inspection and 12/250 patched neurons, which were located within the imaging volume but could not be unambiguously identified, were excluded from further analysis.

In vitro *whole-cell recording*

In vitro recordings were carried out at least 12 hours after the imaging session in 26-42 day old mice. Mice were anesthetized with sodium pentobarbital and transcardially perfused with a cold choline chloride-based solution containing (in mM): 110 choline chloride, 25 $NaHCO_3$, 25 D-glucose, 11.6 sodium ascorbate, 7 $MgCl_2$, 3.1 sodium pyruvate, 2.5 KCl, 1.25 NaH_2PO_4, and 0.5 $CaCl_2$ with $\sim$ 325 mOsm. Visual cortex slices (300–350 μm thickness) were cut coronally on a vibrating blade microtome (VT1200S, Leica Biosystems) with the same choline chloride-based solution bubbled with 95% O_2/5% CO_2. Then, the brain slices were incubated at 34°C for 20–40 min with artificial cerebrospinal fluid (ACSF) solution containing 125 mM NaCl, 2.5 mM KCl, 1 mM $MgCl_2$, 1.25 mM NaH_2PO_4, 2 mM $CaCl_2$, 26 mM $NaHCO_3$, 25 mM D-glucose; osmolarity was adjusted to 315–320 mOsm by adding D-glucose; bubbled with 95% O_2/5% CO_2, pH 7.4. Afterwards, the brain slices were continuously maintained at room temperature before being transferred to the recording chamber.

In vitro imaging during whole-cell recordings was performed with a Scientifica MP-1000 multiphoton imaging microscope and a mode-locked Ti:sapphire laser (Vision-S, Coherent) with a Nikon 16x water-immersion objective (NA 0.8). Scanning and image acquisition were controlled by SciScan (Scientifica) and custom software written in LabVIEW (National Instruments).

Recording pipettes were mounted on remote-controlled motorized micromanipulators (MicroStar, Scientifica). Recording pipettes were made using thick-walled filamentous borosilicate glass capillaries (G150F-4, Warner Instruments) using a horizontal puller (P-1000, Sutter Instrument) adjusted to produce pipette resistance of 7–8 MOhm with a long taper when filled with intracellular solution in 34°C ACSF. The potassium based internal solution contained 5 mM KCl, 115 mM K-gluconate, 10 mM HEPES, 4 mM Mg-ATP, 0.3 mM Na-GTP, 10 mM Na-phosphocreatine, 0.1% w/v Biocytin; osmolarity 290–295 mOsm, pH 7.2. Liquid junction potentials were not corrected.

Whole-cell recordings were carried out at $\sim$34°C using Multiclamp 700B amplifiers (Axon Instruments) and custom-written Matlab software (MathWorks). Up to 6 cells, at least one of which was a tdTomato-expressing PV neuron, were targeted simultaneously. All tdTomato positive cells showed fast-spiking firing profiles during current injection and evoked inhibitory post-synaptic potentials in connected pyramidal cells. Pyramidal neurons were identified based on their regular spiking firing profiles. During 9 out of 35 experiments, the experimenter could target cells based on the correlation of their calcium traces in the *in vivo* imaging dataset. The remaining experiments were carried out blind to the functional properties of the patched neurons.

To reveal both excitatory and inhibitory inputs, pyramidal cells were depolarized to -55– -50 mV by current injection. To test for the presence of synaptic connections, two or five presynaptic spikes were evoked by current injections at 30 Hz in each cell sequentially repeated 20–150 times, while searching for corresponding postsynaptic responses. Recordings with postsynaptic cell series resistances below 35 MOhm were included for analysis.

Models of specific inhibitory and excitatory connectivity

Parameter	Description
N	Number of neurons in the network ($N = 5000$)
w_E, w_I	Total output synaptic weight made by single excitatory and inhibitory neurons, respectively ($w_E, w_I > 0$)
f_E, f_I	Proportion of excitatory and inhibitory neurons in the network ($f_E + f_I = 1$; $f_I = 0.2$)
d_{IE}	Factor defining how much stronger $E \rightarrow I$ synaptic connections are compared with $E \rightarrow E$ ($d_{IE} \geq 1$; $d_{IE} = 2$)
s_E	Proportion of recurrent excitatory synapses that are restricted to be made according to neurons' assigned tuning ($0 \leq s_E \leq 1$)
s_I	Proportion of synapses between excitatory and inhibitory neurons, and recurrent inhibitory synapses, that are restricted to be made according to neurons' assigned tuning ($0 \leq s_I \leq 1$)
κ_{BA}	Concentration parameter controlling synaptic specificity over feature parameter in feature tuning model, for neurons of class A targetting neurons of class B ($\kappa_{EE} = 4$; $\kappa_{EI}, \kappa_{IE}, \kappa_{II} = 0.5$)

Parameters used to build the network models are defined in table above. Briefly, a network of N neurons was defined to contain $N \cdot f_E$ and $N \cdot f_I$ excitatory and inhibitory neurons. Dense connections were made between all potential partners (i.e. synaptic fill factors $h_E, h_I = 1$). Neurons were assigned a preference for two "stimulus features", which determined the strength of synaptic connections between them.

To generate connections between neurons we defined specificity parameters s_E and s_I, which determined what proportion of output synapses from single neurons were made according to specific connection rules. A proportion s_E of total recurrent excitatory synaptic weight was reserved to be made based on the neurons' assigned tuning. The remainder of recurrent excitatory weight was distributed uniformly over the network. The same parameter s_I modulated specific connectivity between excitatory and inhibitory neurons. In these models we assumed that $I \rightarrow E$ and $E \rightarrow I$ synaptic specificity was equal.

For each of $D = 2$ tuning dimensions, neurons were assigned a preferred tuning value $\gamma^{(d)}$, uniformly distributed over $[-\pi, \pi)$. Synaptic connection strength between two neurons i and j was modulated by similarity measured over this tuning parameter. In simulation in panels 5A-D, where the specificity parameters $s_E \geq 0$ and $s_I \geq 0$, the connection strength from a neuron i of class A to a neuron j of class B was given by the function

$$s_{ji} = < \prod_{d=1}^{D} \varphi\left(\gamma_i^{(d)}, \gamma_j^{(d)}, \kappa_{BA}^{(d)}\right) > ,$$

where the circular function $\varphi(\gamma_i, \gamma_j, \kappa)$ defines the tuning of synaptic connections for each stimulus dimension,

$$\varphi(\gamma_i, \gamma_j, \kappa) = \exp\{\kappa_{BA} \cos(\gamma_i - \gamma_j)\},$$

and κ_{BA} is a concentration parameter modulating connections from class A to class B. The notation $<\ >$ indicates the expression within the brackets has been normalized to define a probability density function over target synaptic partners by subtracting the minimum and dividing by the sum for each connection type.

In simulation in Figure 5E, where $s_I < 0$, inhibitory connections were instead defined as

$$s_{ji} = < 1 - \prod_{d=1}^{D} \varphi\left(\gamma_i^{(d)}, \gamma_j^{(d)}, \kappa_{BA}^{(d)}\right) > .$$

The values s_{ji} were composed into the $N \times N$ matrix

$$\mathbf{S} = \begin{bmatrix} \mathbf{S}_{EE} & \mathbf{S}_{EI} \\ \mathbf{S}_{IE} & \mathbf{S}_{II} \end{bmatrix},$$

with the blocks $\mathbf{S}_{BA}$ defining the connections from class A to class B. The synaptic weights connecting neurons were also modulated by specificity parameters, defining a proportion of synapses that were made under the function above. The remainder of synapses were distributed uniformly over the network. The weight matrix for the network was then given in block form by

$$\mathbf{W} = \begin{bmatrix} \mathrm{W_{EE}} & -\mathrm{W_{EI}} \\ \mathrm{W_{IE}} & -\mathrm{W_{II}} \end{bmatrix}, \text{where}$$

$$\mathbf{W}_{EE} = w_E[f_E(|s_E| \cdot \mathbf{S}_{EE}) + (1 - |s_E|)/N]$$

$$\mathbf{W}_{IE} = w_E \cdot d_{IE}[f_I(|s_I| \cdot \mathbf{S}_{IE}) + (1 - |s_I|)/N]$$

$$\mathbf{W}_{EI} = w_I[f_E(|s_I| \cdot \mathbf{S}_{EI}) + (1 - |s_I|)/N]$$

$$\mathbf{W}_{II} = w_I[f_I(|s_I| \cdot \mathbf{S}_{II}) + (1 - |s_I|)/N].$$

Each neuron in the network received a barrage of independent Poisson input spikes at 2400 Hz, designed to mimic ongoing spontaneous activity in cortex. Stimulus input was provided by injecting currents into the excitatory population alone, since input provided directly to the inhibitory population can give rise to suppression of excitatory activity through feed-forward inhibition. Since our goal was to examine recurrent computations, we wished to exclude this feed-forward effect from our analysis. Stimulus preference of inhibitory cells was defined by their connectivity with excitatory neurons. Inputs were tuned according to a similar rule to excitatory connections – input to the ith neuron was defined as

$$\tilde{\mathrm{u}}_{\mathrm{i}} = \frac{\mathrm{u_i} - \min(\mathbf{u})}{\max(\mathbf{u}) - \min(\mathbf{u})}, \text{where}$$

$$\mathrm{u_i} = \prod_{\mathrm{d}=1}^{\mathrm{D}} \varphi\left(\gamma_{\mathrm{i}}^{(\mathrm{d})}, \gamma_{\mathrm{stim}}^{(\mathrm{d})}, \kappa_{\mathrm{EE}}^{(\mathrm{d})}\right),$$

and $\gamma_{stim}^{(d)}$ is value of the dth stimulus feature. The stimulus input was scaled to a maximum of 12 pA and applied for 400 ms at 1 Hz.

To compute response similarity metrics in Figure 5F, the network was stimulated with 16 stimuli spanning both stimulus features, in increments of $\pi/2$ for each dimension. Firing rates were calculated in bins of 250 ms (similar to the *in vivo* imaging rate) and total similarity and correlation of the resulting activity vectors were computed as described above.

Excitatory and inhibitory conductances in Figure 5I were measured across the excitatory population stimulated at their preferred stimulus for the second stimulus feature $\gamma^{(2)}$ by averaging the conductance across the entire stimulus period. Single trial conductance examples Figure 5G are from an excitatory neuron stimulated close to its preferred stimulus ($\gamma_i^{(1)} = \gamma_{stim}^{(1)} - 0.031$, $\gamma_i^{(2)} = \gamma_{stim}^{(2)}$). The correlation of excitatory and inhibitory conductances in Figure 5H was computed based on the entire simulation period in Figures 5A, 5B, and 5D across the excitatory population stimulated at their preferred stimulus for the second stimulus feature $\gamma^{(2)}$ in 0.1 ms increments.

Perturbations of small cohorts of neurons (Figure 6) were performed by first increasing background activity to 3500 Hz per neuron. 11 excitatory neurons (corresponding to 0.275% of the excitatory network) with similar feature tuning $\gamma^{(1)}$ for Feature 1 and matching tuning $\gamma^{(2)}$ for Feature 2 were then stimulated by injecting currents of 200 nA for 10 ms at 10 Hz. Pre-stimulation firing rate in Figure 6C was calculated using 20 ms preceding current injection. Post-stimulation firing rate was calculated using the 50 ms following stimulation onset.

Simulations of integrate and fire spiking neurons with alpha-function conductance-based synapses were performed using Nest 2.12[53] and Matlab (MathWorks). Networks were connected following the weight matrices defined above.

QUANTIFICATION AND STATISTICAL ANALYSIS

Statistical analyses were performed in Matlab. Sample sizes, including numbers of connections and mice, and statistical tests used can be found in the results and figure legends. Confidence intervals were estimated by bootstrap resampling.

Data analysis: preprocessing

Two-photon imaging frames were registered using a phase-correlation algorithm. ROIs were identified based on the mean fluorescence image and their fluorescence time series were extracted. To correct for bleaching and other sources of non-stationarity, we subtracted the running 40th percentile of the fluorescence trace computed in a 500 frame window less its median over the entire trace. Surrounding fluorescence was measured in a ~40 μm radius around each cell, excluding any detected ROIs.

CellPress
OPEN ACCESS

We next sought to remove contaminating neuropil fluorescence from each ROI. To do this, we fit both ROI and surround fluorescence to asymmetric Student-t (ASt) distributions, whose mean was determined by a common neuropil signal contributing to both ROI and surrounding fluorescence, as described in Orsolic et al.[52]. The code and detailed description of the ASt model have been deposited at https://doi.org/10.5281/zenodo.10404602.

To calculate $\Delta F/F_0$, we fit a mixture of two Gaussians to the distribution of neuropil-corrected fluorescence values and used the mean of the smaller Gaussian as an estimate of baseline fluorescence F_0.

Data analysis: responsiveness and selectivity

To identify robustly responsive neurons without making assumptions about the shape of the neurons' tuning curve or the time-courses of their responses, we computed the fraction of variance explained by the visual stimulus:

$$R^2 = 1 - \frac{\text{Var}(f(i,j,t) - \bar{f}(i,t))}{\text{Var}(f(i,j,t))}$$

where $f(i,j,t)$ is the dF/F of the neuron during frame t of the ith stimulus type on the jth trial, and

$$\bar{f}(i,t) = \frac{1}{N_{rep}} \sum_{j=1}^{N_{rep}} f(i,j,t)$$

is the trial average response on frame t of the ith stimulus type, and N_{rep} is the number of trials.

We classified neurons as responsive, if the visual stimulus explained > 15% of the variance of their dF/F responses. Note that while this measure is related to the F-statistic used in ANOVA, our criterion is more stringent than an F-test. While PV− 221/248 ROIs studied in the in vivo/in vitro experiments were significantly responsive by one-way ANOVA ($p < 0.05$), only 155/250 of them passed our variance explained threshold.

To compute the cells' selectivity we first computed the mean dF/F response during the moving phase of the grating (a^{th} to b^{th} frame) for the each stimulus type:

$$r(i,t) = \frac{1}{N_{rep}} \sum_{j=1}^{N_{rep}} f(i,j,t),$$

$$r(i) = \frac{1}{b - a+1} \sum_{t=a}^{b} r(i,t).$$

Neuronal selectivity was defined as the skewness of $\bar{r}(i)$:

$$\gamma = \frac{\frac{1}{N_{stim}} \sum_{i=1}^{N_{stim}} (\bar{r}(i) - \bar{r})^3}{\left(\frac{1}{N_{stim}} \sum_{i=1}^{N_{stim}} (\bar{r}(i) - \bar{r})^2\right)^{3/2}},$$

where $\bar{r} = \frac{1}{N_{stim}} \sum_{i=1}^{N_{stim}} r(i)$ is the mean response across all stimulus types, and N_{stim} is the number of stimulus types.

Data analysis: direction and SF/TF tuning

Parameters of the direction, spatial and temporal frequency fit.

Parameter	Description
b	Response offset
r_{max}	Response at preferred spatial, temporal frequency, and direction, $0 < r_{max} \leq 2 \cdot \max(r_{i,j})$
SF_{pref}, TF_{pref}	Preferred spatial and temporal frequency in octaves, constrained to lie within 1 octave of the range probed in the experiment
α	Orientation of the SF/TF tuning Gaussian, $0^\circ \leq \alpha \leq 90^\circ$
σ_x, σ_y	Tuning width of SF/TF Gaussian, constrained between 0.25 and 4 octaves
σ_{dir}	Width of direction tuning, $0^\circ \leq \sigma_{dir} \leq 180^\circ$
q	Relative magnitude of the response to the null direction, $0 \leq q \leq 1$
θ_{pref}	Preferred direction, $0^\circ \leq \theta_{pref} \leq 360^\circ$

To characterize the direction, spatial and temporal frequency tuning of individual cells, we fit their responses as described in Kim et al.[54]. Single trial responses during the moving phase of the grating

$$r(i,j) = \frac{1}{b - a+1}\sum_{t=a}^{b} f(i,j,t)$$

were modeled as the product of a double Gaussian in direction space and a 2D Gaussian in spatial and temporal frequency space with arbitrary orientation. Parameters of the fit are described in the table above. Let $x(i)$ and $y(i)$ be defined as:

$$\begin{bmatrix} x(i) \\ y(i) \end{bmatrix} = \begin{bmatrix} \cos\alpha & \sin\alpha \\ -\sin\alpha & \cos\alpha \end{bmatrix} \cdot \begin{bmatrix} SF(i) - SF_{pref} \\ TF(i) - TF_{pref} \end{bmatrix}$$

The response $r_{i,j}$ was then modeled as:

$$\hat{r}(i,j) = r_{max}\left[\exp\left\{-\frac{h(\theta(i) - \theta_{pref})}{2\sigma_{dir}^2}\right\} + q\exp\left\{-\frac{h(\theta(i) - \theta_{pref}+\pi)}{2\sigma_{dir}^2}\right\}\right] \times \exp\left\{-\left(\frac{x(i)^2}{2\sigma_x^2} + \frac{y(i)^2}{2\sigma_y^2}\right)\right\} + b$$

where $h(\theta)$ wraps angles onto the interval between 0 and π:

$$h(\theta) = (\min(|\theta|, |\theta+2\pi|, |\theta - 2\pi|))^2$$

We determined the values of the fit parameters using *lsqnonlin* in Matlab by minimizing $\sum_{i=1}^{N_{stim}}\sum_{j=1}^{N_{rep}}(r(i,j) - \hat{r}(i,j))^2$ with the constraints specified in Table 1.

$FWHM_{Dir}$ was calculated as $2.355 \cdot \sigma_{dir}$. If $FWHM_{Dir}$ exceeded 180 degrees, cells were classified as untuned for direction/orientation and excluded from direction selectivity analysis (Figure 2E). $FWHM_{SF}$ and $FWHM_{TF}$ were calculated from tuning curves derived by estimating $\hat{r}(i,j)$ using the equation above with the estimated fit parameters over a range of spatial and temporal frequencies (0.0025 to 2.56 cycles per degree, and 0.125 to 16 Hz). If $FWHM_{SF}$ or $FWHM_{TF}$ exceeded 6 octaves, cells were classified as untuned for spatial or temporal frequency, respectively. Only cells classified as responsive were included in this analysis.

Data analysis: synaptic connectivity

Connected pairs were identified by inspecting all acquired postsynaptic traces. However, as the magnitude of postsynaptic responses often fluctuated following repeated presynaptic stimulation, we limited our analysis of the magnitude of postsynaptic responses to at most the first 20 trials. After subtracting the baseline membrane potential (average Vm in 5 ms preceding presynaptic stimulation), postsynaptic responses were aligned to the time of the presynaptic spike and averaged. Cell pairs where the presence or absence of a connection could not be unambiguously determined were excluded from further analysis. IPSP and EPSP magnitudes were estimated as the minimum or maximum of the mean postsynaptic trace, respectively. In 13/124 PV to pyramidal cell pairs and 7/120 pyramidal to PV cell pairs, where presynaptic stimulation evoked multiple spikes and PSPs overlapped, IPSP and EPSP magnitudes were estimated in the window preceding the onset of the second PSP. Excluding these pairs from the analysis did not affect our conclusions.

For paired-pulse facilitation / depression analyses, the magnitude of the postsynaptic responses to the second pulse was determined from the mean postsynaptic trace as above, subtracting the baseline prior to the second pulse. Cell pairs, where the first pulse evoked multiple presynaptic spikes were excluded from this analysis.

As IPSP and EPSP magnitudes roughly followed a log-normal distribution, we used log-transformed PSP magnitudes to quantify their relationship with each other, as well as response similarity and other response features.

We used the following procedure to test whether the correlation between IPSP and EPSP magnitudes for reciprocally connected Pyr-PV pairs could be explained by variation in slice quality. We first selected PV cells, for which we measured the strength of reciprocal connections with at least 2 pyramidal cells. We then normalized the log-IPSP and log-EPSP magnitudes by their mean for each such PV cell:

$$\mathrm{IPSP}_c^{norm} = \frac{\log_{10}\mathrm{IPSP}_c}{1\Big/n\sum_{i=1}^{n}\log_{10}\mathrm{IPSP}_i}$$

$$\mathrm{EPSP}_c^{norm} = \frac{\log_{10}\mathrm{EPSP}_c}{1\Big/n\sum_{i=1}^{n}\log_{10}\mathrm{EPSP}_i}$$

where IPSP_c and EPSP_c are the magnitudes of connections between the PV cell and the cth pyramidal cell. This is equivalent to normalizing EPSP and IPSP magnitudes using their geometric means. Our conclusions were unchanged if we used their arithmetic means instead (Figure S1F). We quantified the relationship between normalized IPSP and EPSP magnitudes across all recordings as the Pearson product moment correlation coefficient:

$$R_{EI} = \frac{\text{cov}(\text{IPSP}^{norm}, \text{EPSP}^{norm})}{\sigma_{\text{IPSP}^{norm}}\sigma_{\text{EPSP}^{norm}}}$$

To assess the statistical significance of this relationship, we randomly permuted IPSP magnitudes for each PV cell, destroying any relationship between IPSP and EPSP strength, and recalculated the correlation coefficient. The P-value was estimated as the proportion of reshuffled samples, where the absolute value of the correlation exceeded the one observed empirically.

To quantify the time courses of postsynaptic responses, we fit average postsynaptic traces with a multiexponential model:

$$V(t) = V_{max}(1 - \exp(-t/\tau_r))^p \exp(-t/\tau_d)$$

V_{max} sets the connection amplitude, τ_r and τ_d are the time constants of the rise and decay of the responses, respectively, and the power parameter p adjusts the shape of the onset. Optimal parameters were determined by minimizing the sum square error of the fit using *fit* in Matlab over 100 iterations with different starting parameter values. To avoid artefacts of presynaptic stimulation, we excluded the first 5 ms of the response from the fit.

Data analysis: relating synaptic connectivity and *in vivo* responses

Cell pairs profiled in vitro were only included in the analysis in Figure 4 if both cells were located within the imaging volume and could be reliably identified. Of the 157 PV cell to pyramidal cell connections and 164 pyramidal cell to PV cell connections, 119 and 118 respectively passed this criterion. Since fluorescence cross-talk between overlapping ROIs would inflate our estimate of response similarity, we excluded cell pairs separated by $< 10\ \mu$m in the same or adjacent imaging planes. Finally, we restricted our analysis to pairs where the pyramidal cell was visually responsive and passed the signal variance threshold of 15%. This dataset included 67 PV cell to pyramidal cell pairs (from 22 mice) and 64 pyramidal cell to PV cell pairs (from 23 mice), of which 52 (from 18 mice) and 40 (from 19 mice) respectively were connected.

To compute total response similarity between pairs of ROIs, we computed the dot product of their dF/F_0 traces normalized by the product of their norms for each imaging segment, and used the mean of these values:

$$sim(f,f') = \frac{1}{N_{rep}}\sum_{j=1}^{N_{rep}}\frac{\sum_i\sum_t f(i,j,t)f'(i,j,t)}{\left(\sum_i\sum_t f(i,j,t)^2\sum_i\sum_t f'(i,j,t)^2\right)^{1/2}}$$

where $f(i,j,t)$ and $f'(i,j,t)$ are dF/F_0 traces of the two cells for the ith stimulus type on the jth trial. To compute response similarity, we used the trial-average responses $r(i,t)$ and $r'(i,t)$:

$$sim(r,r') = \frac{\sum_i\sum_t r(i,t)r'(i,t)}{\left(\sum_i\sum_t r(i,t)^2\sum_i\sum_t r'(i,t)^2\right)^{1/2}}$$

Similarity metrics for individual visual features were computed as follows:

$$\begin{aligned}
\Delta\text{TF} &= \left|TF_{pref} - TF_{pref}'\right| \\
\Delta\text{SF} &= \left|SF_{pref} - SF_{pref}'\right| \\
\Delta\text{Speed} &= \left|(TF_{pref} - SF_{pref}) - (TF_{pref}' - SF_{pref}')\right| \\
\Delta\text{Ori} &= 90^\circ - \left|\left|\varphi - \varphi'\right| - 90^\circ\right|
\end{aligned}$$

where φ and φ' are the preferred orientations of each cell, θ_{pref} mod 180°.

Additional response similarity metrics in Figures S4–S6 were computed as follows:

$$\text{Signal resp. similarity time-averaged} = \frac{\sum_i \bar{r}(i)\bar{r}'(i)}{\left(\sum_i \bar{r}(i)^2 \sum_i \bar{r}'(i)^2\right)^{1/2}}$$

$$\text{Total correlation} = \frac{1}{N}\sum_{j=1}^{N}\frac{\text{cov}\left(f_{i,j}(t), f_{i,j}'(t)\right)}{\sigma_{f_{i,j}(t)}\sigma_{f_{i,j}'(t)}}$$

$$\text{Signal correlation} = \frac{\text{cov}(r(i,t), r'(i,t))}{\sigma_{r(i,t)}\sigma_{r'(i,t)}}$$

SF, TF, and direction tuning correlations were each computed in a similar manner, after first averaging $\bar{r}(i)$ across the other two stimulus dimensions.

The relationship between response similarity and connection probability was quantified using logistic regression. The relationship between response similarity or other response metrics and PSP magnitude was quantified as the Pearson product moment correlation coefficient of the functional similarity metric and log-transformed PSP magnitude.

Partial correlations controlling for distance were calculated using *partialcorr* in Matlab, computing the Pearson correlation coefficient of residuals of linear regression of similarity metrics and log-PSP magnitude against distance.

Neuron, Volume *112*

Supplemental information

Functional specificity of recurrent inhibition

in visual cortex

Petr Znamenskiy, Mean-Hwan Kim, Dylan R. Muir, M. Florencia Iacaruso, Sonja B. Hofer, and Thomas D. Mrsic-Flogel

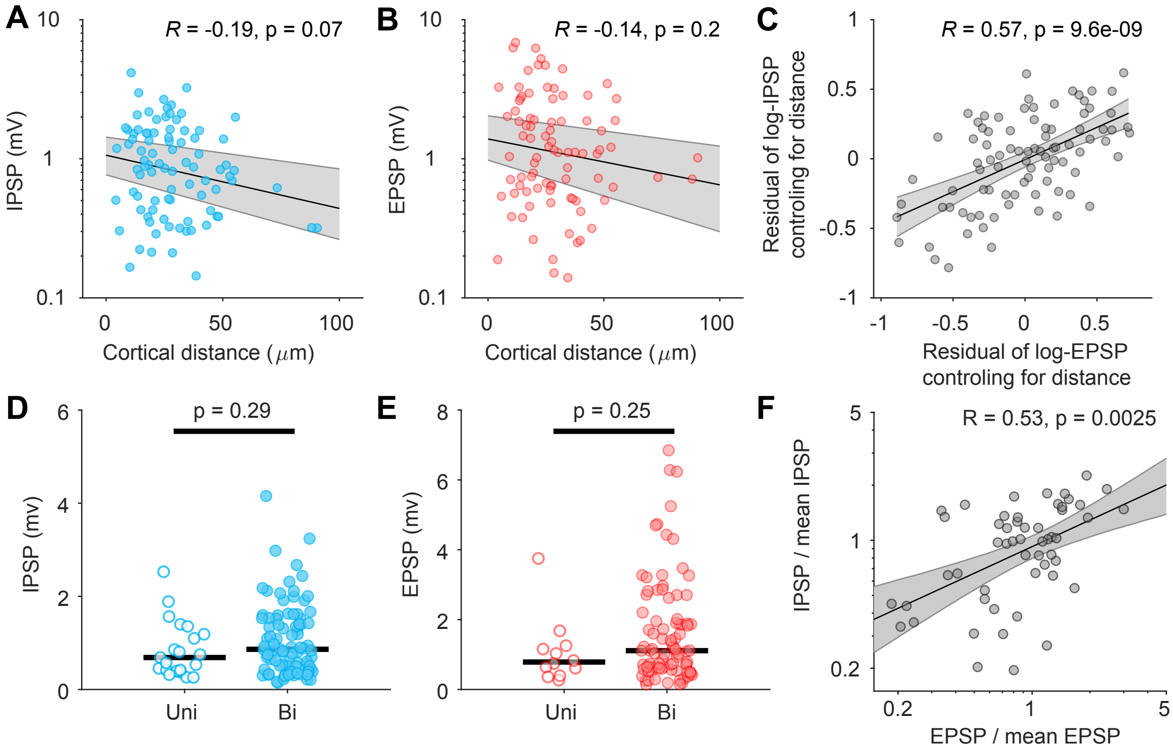

Figure S1: **Distance and slice quality do not account for the correlation between EPSP and IPSP magnitude for reciprocally connected PV+ / pyramidal cell pairs.** Related to Figure 1. (**A–B**) Relationship between cortical distance and IPSP (panel **A**) or EPSP (panel **b**) magnitude. (**C**) EPSP and IPSP magnitudes remain correlated after controlling for distance. (**D–E**) No significant difference in the strength of unidirectional and bidirectional inhibitory (**D**, 21 and 88 uni- and bi-directional connections, respectively) and excitatory connections (**E**, 12 and 88 connections). Black lines - medians; p-values – ranksum test. (**F**) Correlation between EPSP and IPSP magnitude persists after controlling for slice quality by normalizing each by the arithmetic mean of EPSP/IPSP strength in the recording.

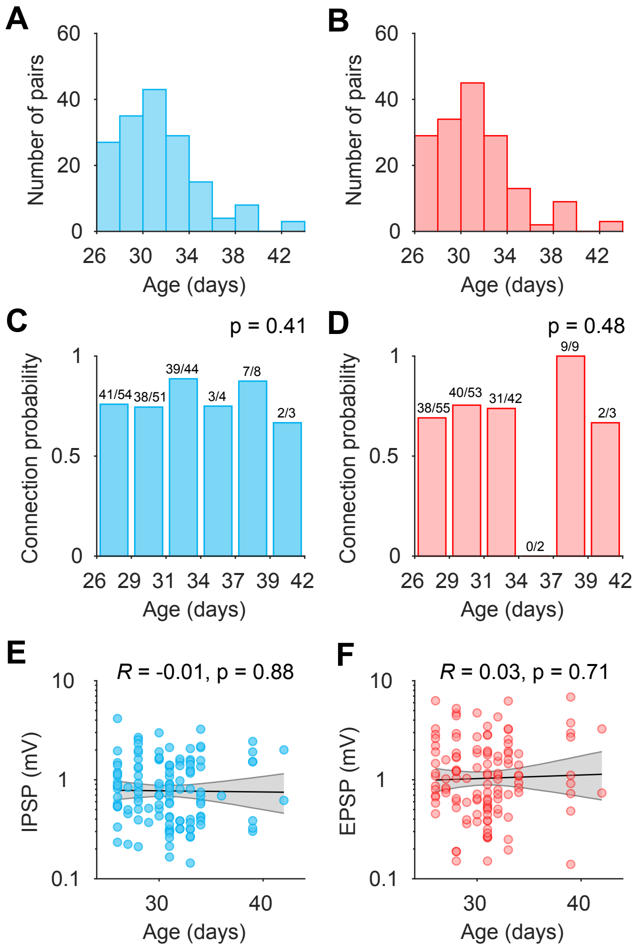

Figure S2: **Differences in mouse age do not account for variability in synaptic strength of PV+ neurons.** Related to Figure 1. (**A–B**) Mouse age at the time of recording for inhibitory (**A**) and excitatory (**B**) connections. (**C–D**) Frequency of inhibitory (**C**) and excitatory (**D**) connections does not depend on mouse age across the ages tested. (**E–F**) Strength of inhibitory (**A**) and excitatory (**B**) connections does not depend on mouse age across the ages tested.

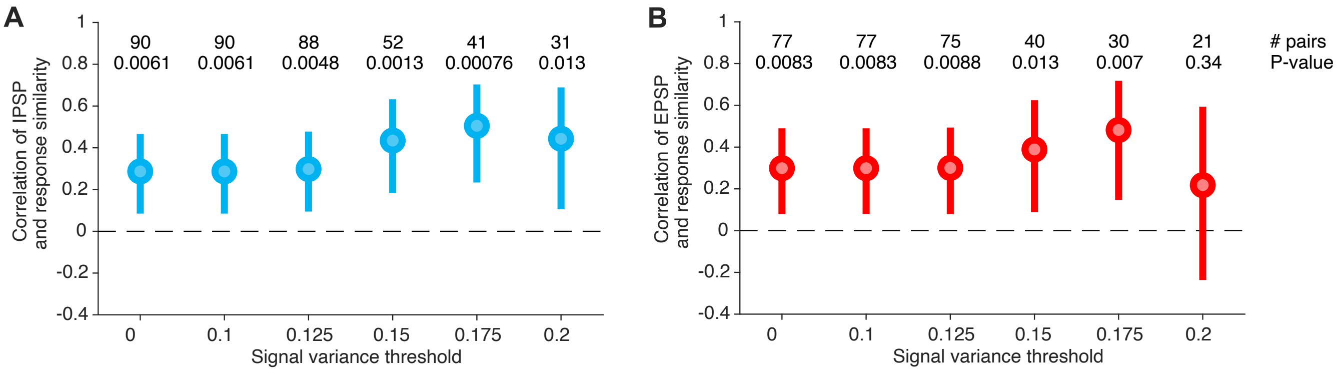

Figure S3: **Relationship between response similarity and connection strength is robust to choice of responsiveness threshold.** Related to Figure 4. Correlation between response similarity and inhibitory (panel **A**) or excitatory (panel **B**) connection strength across a range of responsiveness cut-offs for pyramidal neurons. Error bars are 95% confidence intervals.

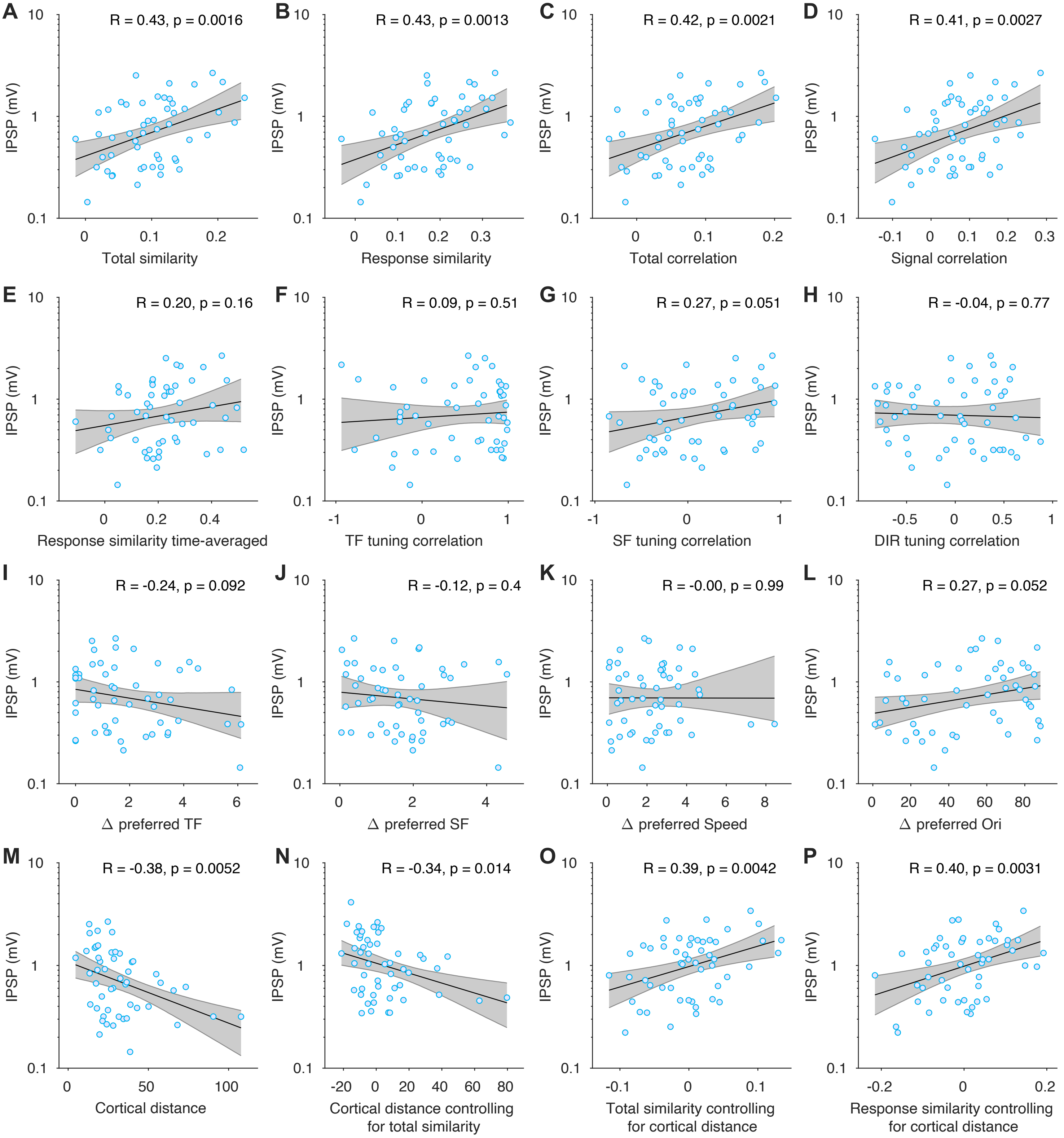

Figure S4: **Post-hoc analysis of relationships between IPSP magnitude and the following response similarity metrics.** Continued on the following page.

Figure S4: **Post-hoc analysis of relationships between IPSP magnitude and the following response similarity metrics.** Related to Figure 4. (**A**) Total response similarity (as in Figure 2). (**B**) Response similarity. (**C**) Total correlation. (**D**) Signal correlation. (**E**) Response similarity, computed over time-averaged responses to moving phase of the grating. (**F**) Correlation of temporal frequency tuning curves, computed from responses to the moving phase of the grating after averaging across spatial frequencies, directions, and time. (**G**) Correlation of spatial frequency tuning curves, computed from responses to the moving phase of the grating after averaging across temporal frequencies, directions, and time. (**H**) Correlation of direction tuning curves, computed from responses to the moving phase of the grating after averaging across temporal and spatial frequencies, and time. (**I**) Absolute difference of preferred temporal frequencies, estimated from model fit. (**J**) Absolute difference of preferred spatial frequencies, estimated from model fit. (**K**) Absolute difference of preferred speed, estimated from model fit. (**L**) Absolute difference of preferred orientations, estimated from model fit. (**M**) Distance between cell pairs in the cortical plane. (**N**) Residuals of linear regression of log-IPSP and cortical distance against total response similarity. (**O**) Residuals of linear regression of log-IPSP and total response similarity against cortical distance. (**P**) Residuals of linear regression of log-IPSP and response similarity against cortical distance.

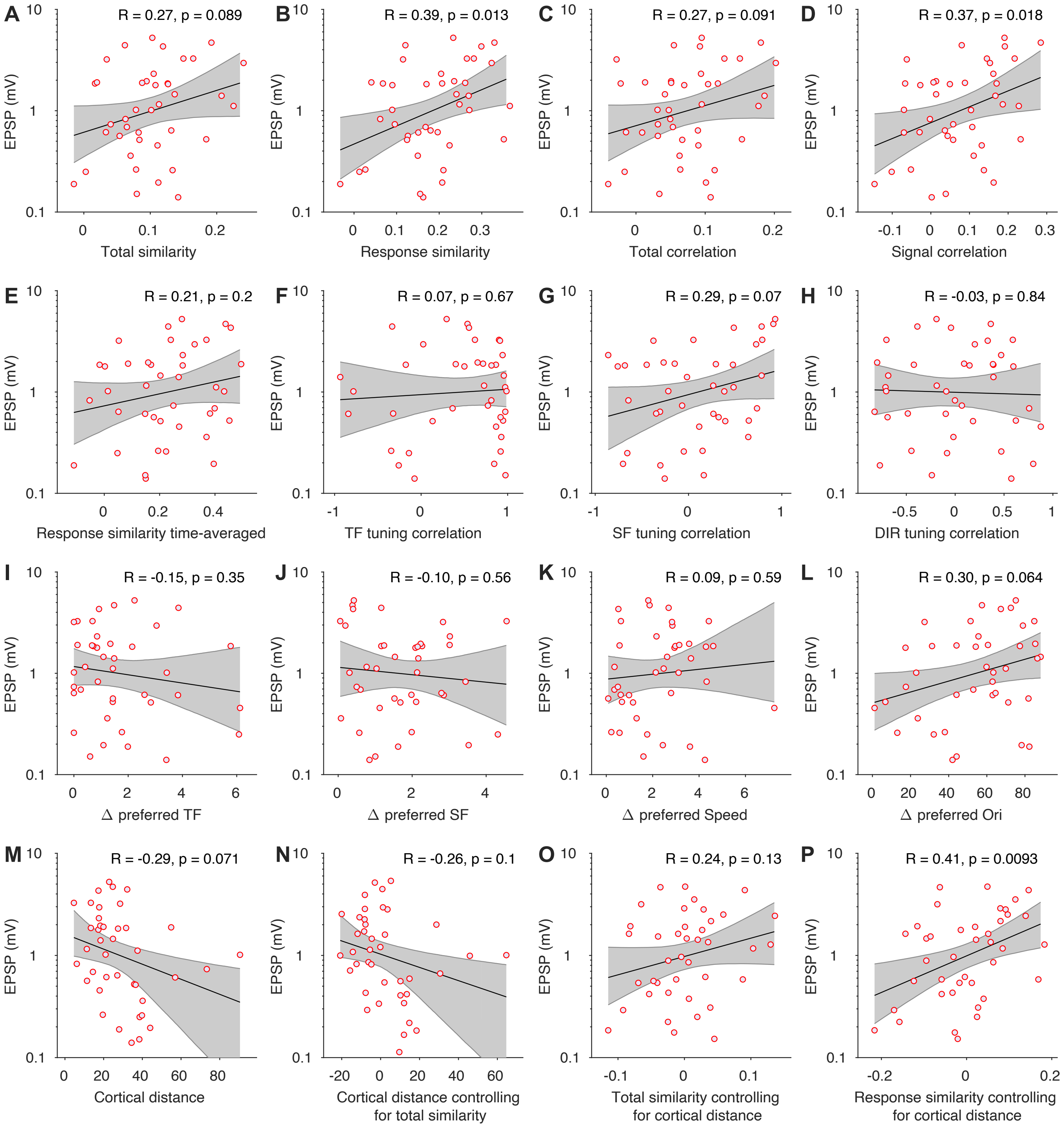

Figure S5: **Post-hoc analysis of relationships between EPSP magnitude and individual response similarity metrics.** Related to Figure 4.

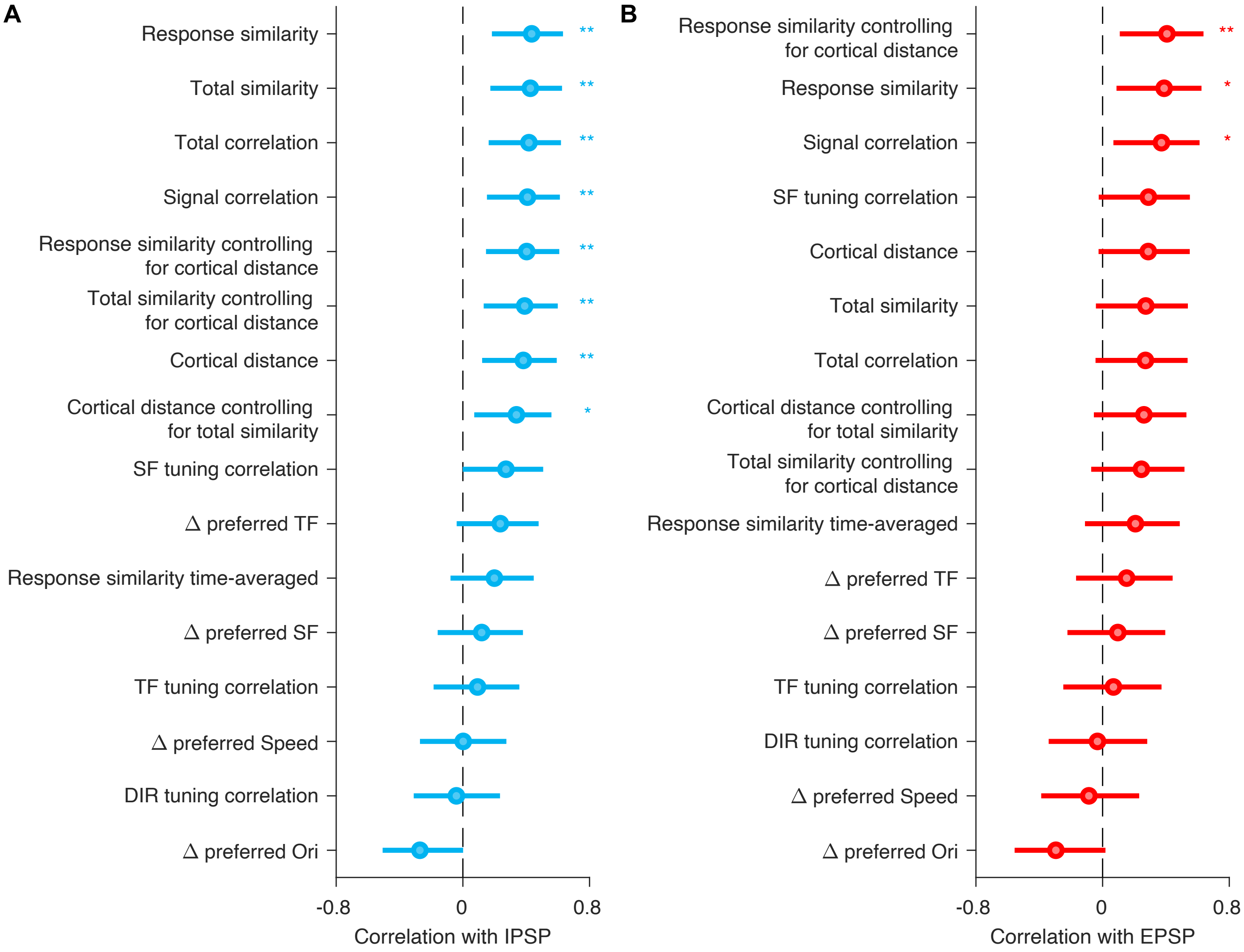

Figure S6: **Summary of relationships between connection strength and response similarity metrics from figures S5–6.** Related to Figure 4. Error bars are 95% confidence intervals. **: $p < 0.01$, *: $p < 0.05$.

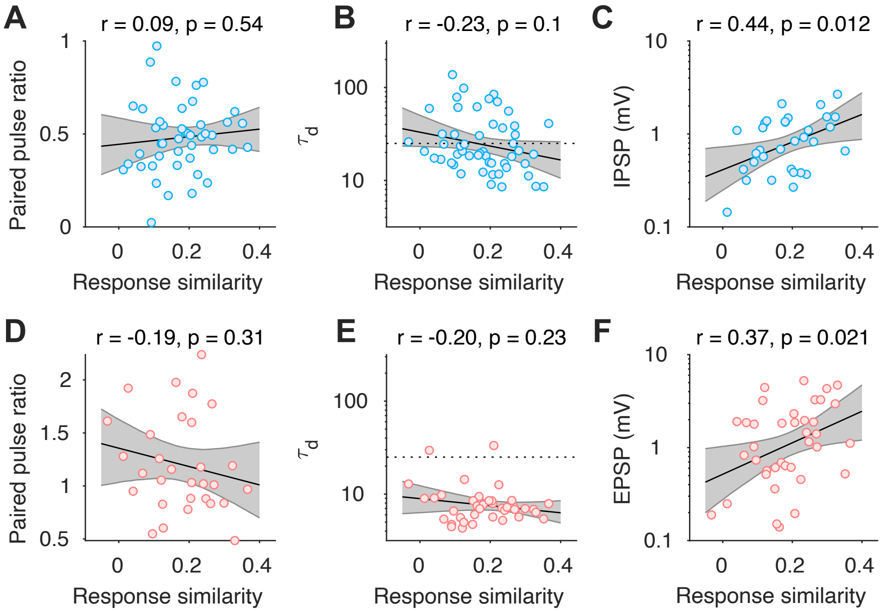

Figure S7: **Short-term dynamics of PV+ neuron connections.** Related to Figure 4. (**A**) Short-term dynamics of inhibitory connections, quantified as paired pulse ratios at 30 Hz, do not depend on response similarity (45 pairs). (**B**) The decay timecourse of inhibitory connections does not depend on response similarity. (**C**) The relationship between IPSP magnitude and response similarity persists when analysis is restricted to connections with rapidly decaying responses ($\tau_d < 25$ ms, dashed line in panel **B**). (**D**) Short-term dynamics of excitatory connections do not depend on response similarity (30 pairs). (**E**) The decay timecourse of excitatory connections does not depend on response similarity. (**F**) The relationship between EPSP magnitude and response similarity persists when analysis is restricted to connections with rapidly decaying responses.
