# Supplementary material for: Functional specificity of recurrent inhibition in visual cortex
Source: Neuron. Author manuscript; Available in PMC 2025 Nov 4. (PMC7618320; doi:10.1016/j.neuron.2023.12.013)
Supplement: Supplementary Material [file EMS209709-supplement-Supplementary_Material.zip › 1-s2.0-S0896627323009728-mmc1.pdf]

Neuron, Volume *112*

Supplemental information

Functional specificity of recurrent inhibition in visual cortex

Petr Znamenskiy, Mean-Hwan Kim, Dylan R. Muir, M. Florencia Iacaruso, Sonja B. Hofer, and Thomas D. Mrsic-Flogel

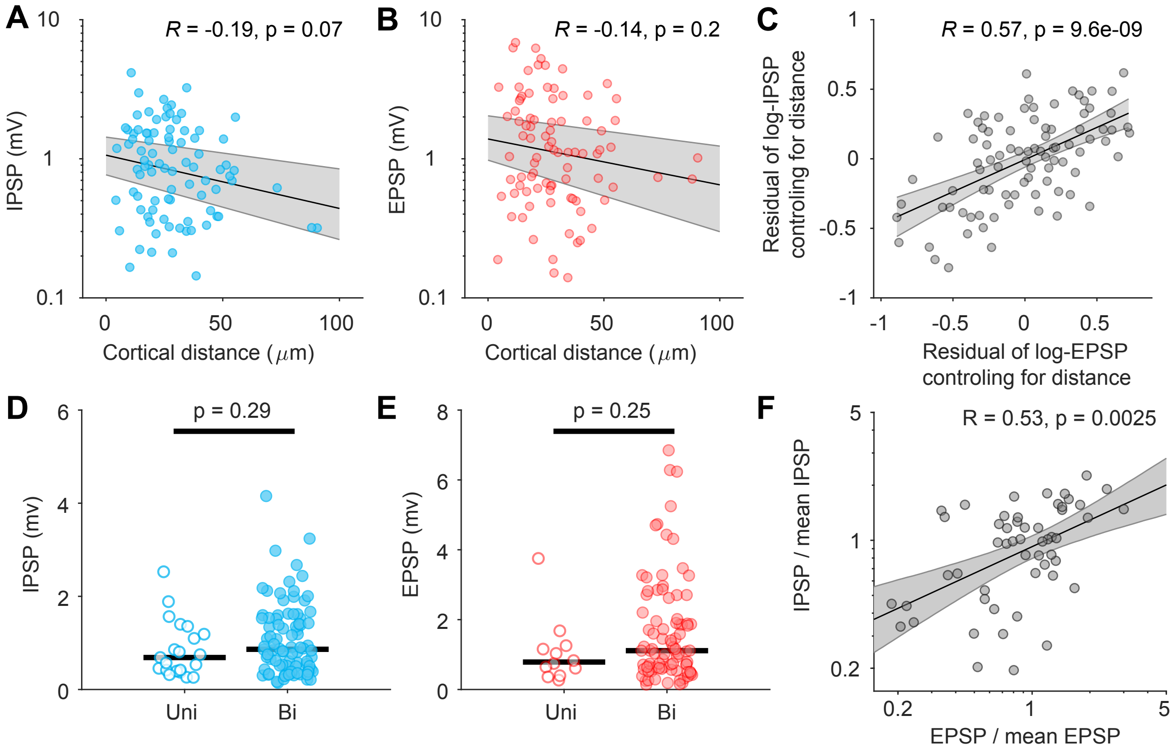

Figure S1: **Distance and slice quality do not account for the correlation between EPSP and IPSP magnitude for reciprocally connected PV+ / pyramidal cell pairs.** Related to Figure 1. (**A–B**) Relationship between cortical distance and IPSP (panel **A**) or EPSP (panel **b**) magnitude. (**C**) EPSP and IPSP magnitudes remain correlated after controlling for distance. (**D–E**) No significant difference in the strength of unidirectional and bidirectional inhibitory (**D**, 21 and 88 uni- and bi-directional connections, respectively) and excitatory connections (**E**, 12 and 88 connections). Black lines - medians; p-values – ranksum test. (**F**) Correlation between EPSP and IPSP magnitude persists after controlling for slice quality by normalizing each by the arithmetic mean of EPSP/IPSP strength in the recording.

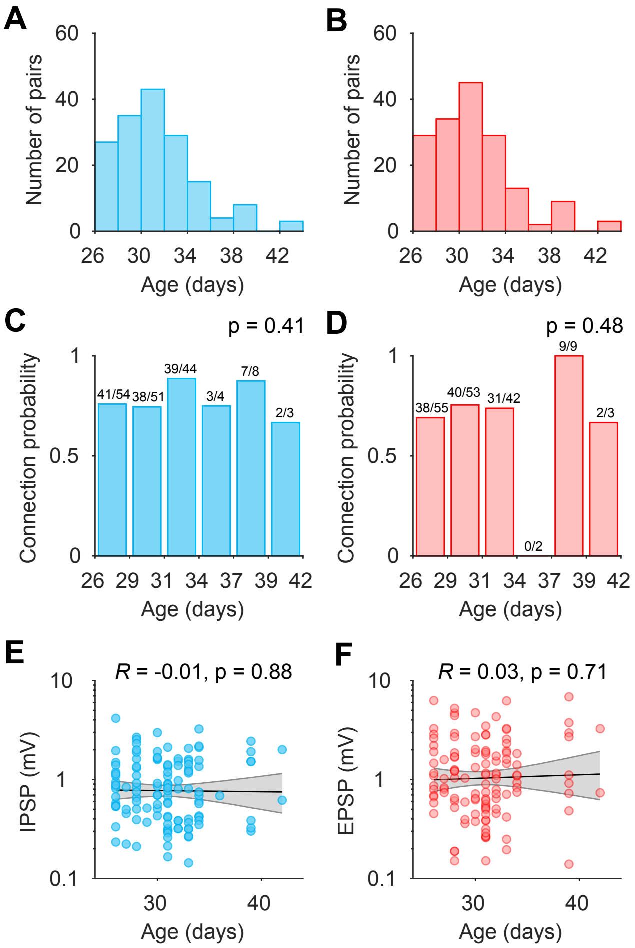

Figure S2: **Differences in mouse age do not account for variability in synaptic strength of PV+ neurons.** Related to Figure 1. (**A–B**) Mouse age at the time of recording for inhibitory (**A**) and excitatory (**B**) connections. (**C–D**) Frequency of inhibitory (**C**) and excitatory (**D**) connections does not depend on mouse age across the ages tested. (**E–F**) Strength of inhibitory (**A**) and excitatory (**B**) connections does not depend on mouse age across the ages tested.

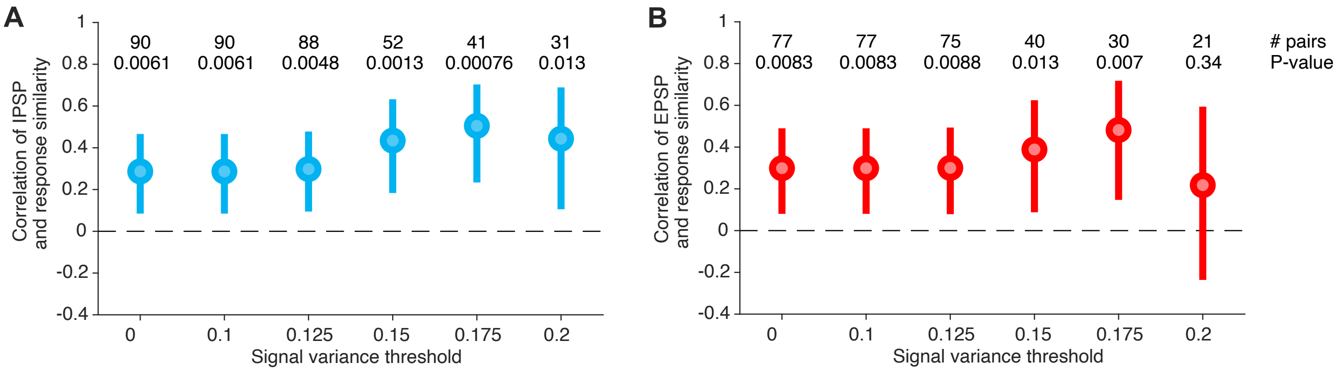

Figure S3: **Relationship between response similarity and connection strength is robust to choice of responsiveness threshold.** Related to Figure 4. Correlation between response similarity and inhibitory (panel **A**) or excitatory (panel **B**) connection strength across a range of responsiveness cut-offs for pyramidal neurons. Error bars are 95% confidence intervals.

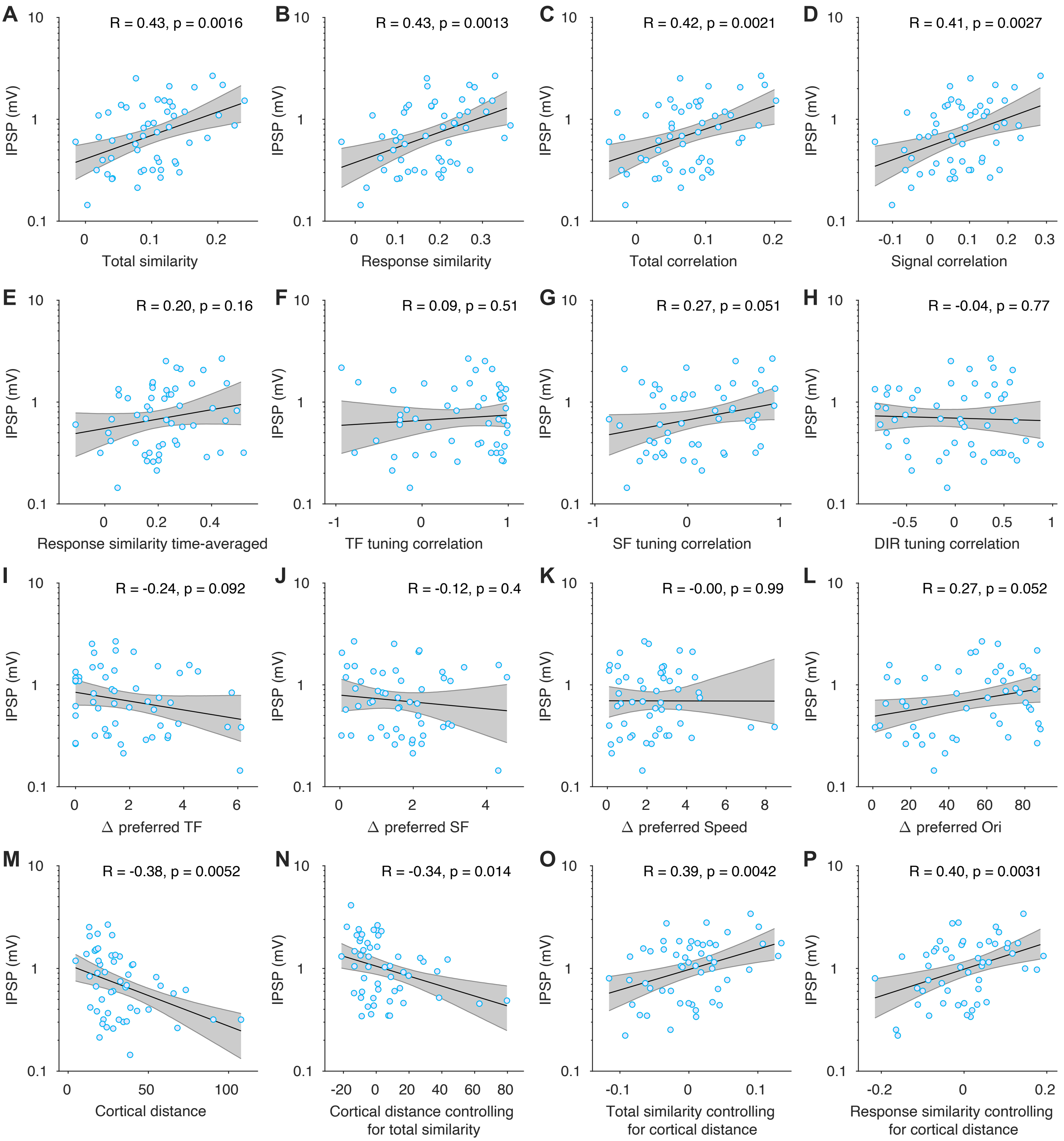

Figure S4: **Post-hoc analysis of relationships between IPSP magnitude and the following response similarity metrics.** Continued on the following page.

Figure S4: **Post-hoc analysis of relationships between IPSP magnitude and the following response similarity metrics.** Related to Figure 4. (**A**) Total response similarity (as in Figure 2). (**B**) Response similarity. (**C**) Total correlation. (**D**) Signal correlation. (**E**) Response similarity, computed over time-averaged responses to moving phase of the grating. (**F**) Correlation of temporal frequency tuning curves, computed from responses to the moving phase of the grating after averaging across spatial frequencies, directions, and time. (**G**) Correlation of spatial frequency tuning curves, computed from responses to the moving phase of the grating after averaging across temporal frequencies, directions, and time. (**H**) Correlation of direction tuning curves, computed from responses to the moving phase of the grating after averaging across temporal and spatial frequencies, and time. (**I**) Absolute difference of preferred temporal frequencies, estimated from model fit. (**J**) Absolute difference of preferred spatial frequencies, estimated from model fit. (**K**) Absolute difference of preferred speed, estimated from model fit. (**L**) Absolute difference of preferred orientations, estimated from model fit. (**M**) Distance between cell pairs in the cortical plane. (**N**) Residuals of linear regression of log-IPSP and cortical distance against total response similarity. (**O**) Residuals of linear regression of log-IPSP and total response similarity against cortical distance. (**P**) Residuals of linear regression of log-IPSP and response similarity against cortical distance.

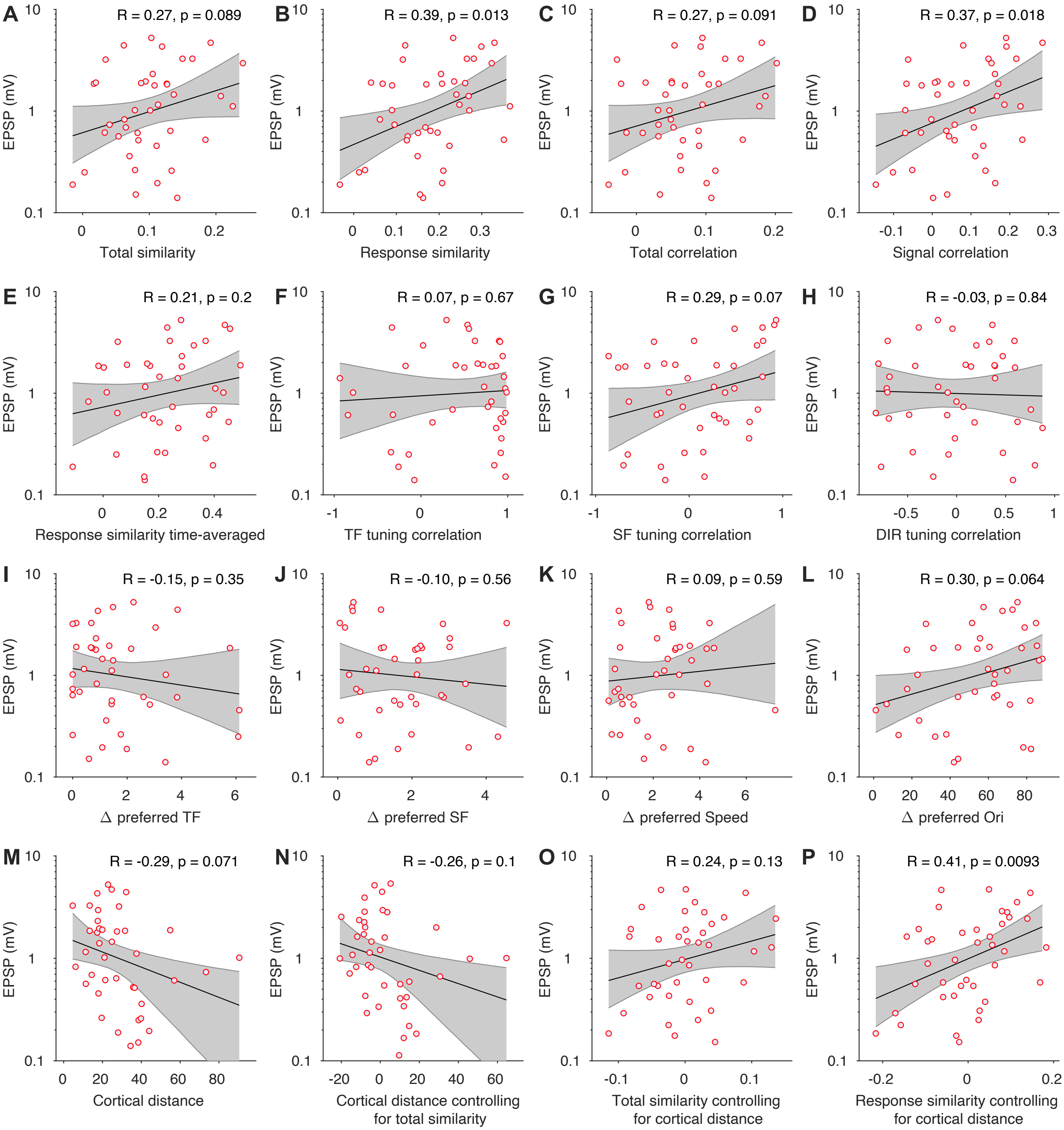

Figure S5: **Post-hoc analysis of relationships between EPSP magnitude and individual response similarity metrics.** Related to Figure 4.

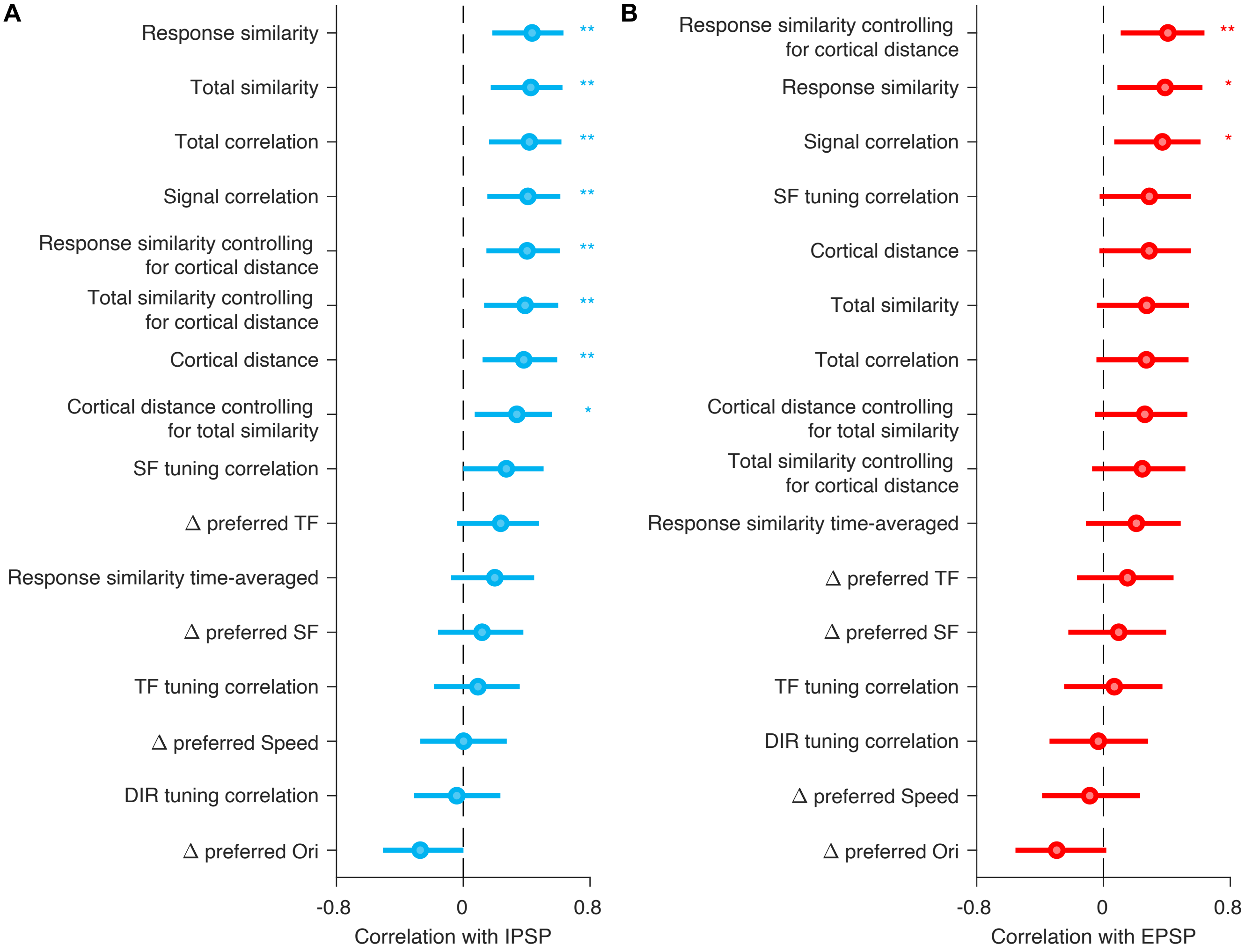

Figure S6: **Summary of relationships between connection strength and response similarity metrics from figures S5–6.** Related to Figure 4. Error bars are 95% confidence intervals. **: $p < 0.01$, *: $p < 0.05$.

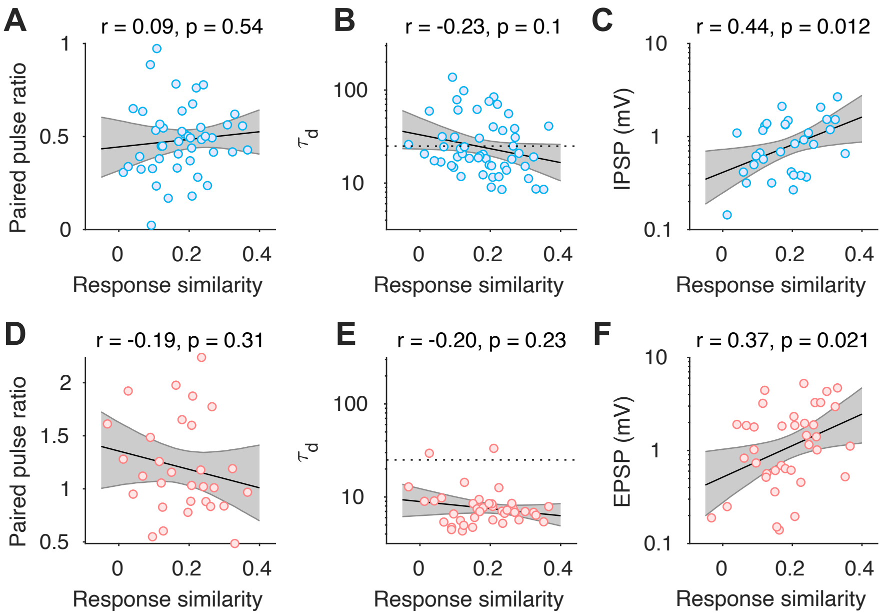

Figure S7: **Short-term dynamics of PV+ neuron connections.** Related to Figure 4. (**A**) Short-term dynamics of inhibitory connections, quantified as paired pulse ratios at 30 Hz, do not depend on response similarity (45 pairs). (**B**) The decay timecourse of inhibitory connections does not depend on response similarity. (**C**) The relationship between IPSP magnitude and response similarity persists when analysis is restricted to connections with rapidly decaying responses ($\tau_d < 25$ ms, dashed line in panel **B**). (**D**) Short-term dynamics of excitatory connections do not depend on response similarity (30 pairs). (**E**) The decay timecourse of excitatory connections does not depend on response similarity. (**F**) The relationship between EPSP magnitude and response similarity persists when analysis is restricted to connections with rapidly decaying responses.
